# A Novel Joint Spatial-Code Clustered Interference Alignment Scheme for Large-Scale Wireless Sensor Networks

**DOI:** 10.3390/s150101964

**Published:** 2015-01-16

**Authors:** Zhilu Wu, Lihui Jiang, Guanghui Ren, Nan Zhao, Yaqin Zhao

**Affiliations:** 1 School of Electronics and Information Engineering, Harbin Institute of Technology, Harbin 150001, China; E-Mails: jianglihui@hit.edu.cn (L.J.); rgh@hit.edu.cn (G.R.); yaqinzhaohit@gmail.com (Y.Z.); 2 School of Information and Communication Engineering, Dalian University of Technology, Dalian 116024, China; E-Mail: zhaonan@dlut.edu.cn

**Keywords:** wireless sensor networks, interference alignment, clustering, pseudo noise (PN) code, spatial-code domain, bit error rate (BER), spread spectrum

## Abstract

Interference alignment (IA) has been put forward as a promising technique which can mitigate interference and effectively increase the throughput of wireless sensor networks (WSNs). However, the number of users is strictly restricted by the IA feasibility condition, and the interference leakage will become so strong that the quality of service will degrade significantly when there are more users than that IA can support. In this paper, a novel joint spatial-code clustered (JSCC)-IA scheme is proposed to solve this problem. In the proposed scheme, the users are clustered into several groups so that feasible IA can be achieved within each group. In addition, each group is assigned a pseudo noise (PN) code in order to suppress the inter-group interference via the code dimension. The analytical bit error rate (BER) expressions of the proposed JSCC-IA scheme are formulated for the systems with identical and different propagation delays, respectively. To further improve the performance of the JSCC-IA scheme in asymmetric networks, a random grouping selection (RGS) algorithm is developed to search for better grouping combinations. Numerical results demonstrate that the proposed JSCC-IA scheme is capable of accommodating many more users to communicate simultaneously in the same frequency band with better performance.

## 1. Introduction

Wireless sensor networks (WSNs) have attracted wide attention in recent years, and have been applied to the areas of environmental monitoring, remote healthcare assistance, battlefield surveillance, intelligent homes, industrial control, *etc.* [1]. In particular, there is an increasing demand for WSNs to be capable of producing multimedia information such as image and video. In addition, the limited battery capacity of the sensors imposes strict constraints on the energy consumption of WSNs. Therefore, high data rates and high energy efficient transmission methods are required by modern WSNs. The multiple-input multiple-output (MIMO) technique represents a promising method which can significantly increase the system throughput and energy efficiency [2], therefore, MIMO has become a prospective communication scheme for WSNs.

Although MIMO exhibits significant advantages in terms of sum rate and energy efficiency, it is still limited by the interference from the unintended users, especially when there are plenty of users. Interference alignment (IA) is a promising technique which can effectively mitigate the interference in the MIMO interference channel, where different users might transmit messages simultaneously via the same frequency band. The main idea of IA is to confine the interference to one half of the signal subspaces, and to recover the desired signal through the other half that is free from interference by designing proper precoding and decoding matrices [3]. Cadambe and Jafar have come to the astonishing conclusion that the sum rate of the MIMO interference channel can increase proportionally with the number of users by applying IA in the high signal-to-noise ratio (SNR) regime [4]. The degrees of freedom (DoFs) of the MIMO interference channel were further studied theoretically in [5], which showed that IA can achieve the maximal DoFs. In addition, the conclusions above were verified experimentally over the measured MIMO orthogonal frequency division-multiplexing (OFDM) channels in [6], and the viability of IA was substantiated. Due to its excellent performance in suppressing interference and increasing the throughput, IA has been applied to relay networks [7], cognitive radio [8], *ad-hoc* networks [9], *etc.* In particular, Pandey *et al.* have validated the feasibility of IA in sensor networks in [10].

Although IA can effectively suppress the interference, the number of users *K* in the networks with *M* transmitting antennas, *N* receiving antennas and *d* DoFs is still limited by the IA feasibility constraint, *i.e.*, *K* ≤ (*M* + *N*)/*d* − 1 [11]. In other words, the scale of the antennas should become larger when the number of users in the networks increases. Unfortunately, the number of antennas is strictly restricted by many limitations such as the equipment volume, power consumption and price. When the amount of users exceeds the IA feasibility constraint, the interference leakage will become so strong that the quality of service (QoS) will degrade significantly and may become unacceptable. To the best of our knowledge, most of the current works on IA were conducted under the assumption that the IA feasibility condition is satisfied, and the methods herein may not perform well when such a constraint cannot be met. Therefore, we focus on this problem in the large-scale networks with limited antennas, where the number of users exceeds that IA can accept.

The idea of clustering [12,13] may provide an alternative approach to address this problem. The users in the networks are divided into several small groups, and IA is applied to each group independently. Halima and Saadani [12] proposed a clustered IA scheme by time division multiplexing. Although it can accommodate more users, real-time transmission cannot be guaranteed when there are plenty of users. A scheduled clustered IA for the asymmetric interference networks was proposed by Chen and Cheng in [13]. Unfortunately, this method cannot be applied to the conventional symmetric interference channel.

Against the deficiencies of the current IA systems above, we propose a novel clustered IA scheme to address the problem of strong interference in the large-scale MIMO networks with limited antennas. The main contributions of this paper may be summarized as follows:

(1) A joint spatial-code clustered (JSCC)-IA scheme is proposed, where the users are partitioned into several small groups. In each cluster, the intra-group interference is eliminated by the traditional spatial IA, and the inter-group interference is suppressed by the orthogonality of the pseudo noise (PN) codes assigned to each group. It is well known that the spread spectrum technique serves as an effective anti-interference approach with the advantage of good confidentiality. Therefore, the proposed IA scheme may serve as a more viable alternative approach to mitigate the interference in large-scale networks.
(2) The analytical expressions of the bit error rate (BER) for the proposed JSCC-IA scheme are formulated in the situations of identical and different propagation delays, respectively. To the best of our knowledge, many of the existing literatures investigate the performance of IA from the perspectives of achievable rate or DoFs, while only a few analyze the error performance. As larger sum rate cannot guarantee better QoS, BER is employed as the performance measurement in this paper.
(3) Both of the symmetric and asymmetric networks are considered, and a random grouping selection (RGS) algorithm is proposed to search for better grouping combinations for the asymmetric networks. The RGS algorithm can further improve the QoS of the JSCC-IA scheme, and provides a good tradeoff between the computational complexity and performance improvement on BER and received SINR.

The rest of the paper is organized as follows: Section 2 presents the related work. Section 3 introduces the system model of a complete binary phase shift keying (BPSK) transmission system in the *K*-user MIMO interference channel. The JSCC-IA scheme is proposed in Section 4, and its mechanism and error performance are analyzed. In Section 5, the random grouping selection algorithm is developed for the asymmetric networks. Simulation results are provided in Section 6, followed by the conclusion and future work in Section 7.

Notation: **A**^T^, **A**^H^, ‖**A**‖, real(**A**), sign(**A**) and **A**(*i*,*j*) represent the transpose, the conjugate transpose, the Frobenius norm, the real part, the sign and the element located on the *i*-th row and the *j*-th column of matrix **A**, respectively. **A***_k_*,*_j_* denotes the *j*-th column of matrix **A***_k_*. **E**[•], var[•] and ⌈ •⌉ are the expected value, the variance and the ceiling operators, respectively. We employ R, C, and C*^M^*^×^*^N^* to represent the spaces of the real value, the complex value and the *M* × *N* complex matrices, respectively. The notation rect*_T_*(*t*) indicates the unit-amplitude rectangular function with non-zero duration of *T*. CN(μ, σ^2^) represents a Gaussian distribution with expected value of μ and variance of σ^2^. The notation (*M* × *N*, *d*)*^K^* means a *K*-user MIMO interference channel where each user transmits *d* data streams via *M* transmitting antennas and *N* receiving antennas.

## 2. Related Work

In this section, we present a further survey on the application of the MIMO technique in the WSNs. Moreover, some key issues on IA are further investigated. As shown in the Introduction, the MIMO technique is one promising method to improve the performance of WSNs. The introduction of multi-antenna transmission to WSNs can further explore the channel capacity. A threshold-based medium access control strategy was developed in [14], which can exploit the diversity gain of the MIMO WSNs. Since the energy consumption of sensor nodes plays a critical role on their lifetime, it is important to increase the energy efficiency to prolong the endurance of the WSNs. Recently, a large number of researchers have employed the MIMO technique to achieve energy efficient WSNs. Yuan *et al.* [15] developed a multi-hop virtual MIMO (VMIMO) scheme to improve the energy efficiency and QoS of the WSNs. The cooperative MIMO and data-aggregation techniques were leveraged to jointly reduce the energy consumption of the sensor nodes in [16]. The random WSNs using cooperative MIMO technique were considered in [17], where packet error rate, nodes active rate and energy consumption were jointly analyzed. Park and Jeong [18] analyzed the performances of two kinds of VMIMO schemes in the WSNs, *i.e.*, space-time block coding and spatial multiplexing, and developed a new switching algorithm between them to achieve energy efficient transmission. In addition, relay communication has been cooperated with the MIMO technique to further enhance the performance of WSNs. A multi-hop hybrid VMIMO scheme was developed in [19], where a route with minimum energy consuming was designed, while satisfying the given data rate and BER constraints. In addition, a number of testbeds for WSNs have been developed such as EasiTest, Emstar, Kansei and MoteLab, which can facilitate the development of the advanced WSNs [20].

Although the MIMO technique has shown bright prospects in WSNs, many problems still exist in this systems. As the scale of WSNs becomes larger and larger, the interference becomes a severe impediment to the increasing demand for communications. Against this background, IA has emerged as a promising technology which can effectively eliminate the interference.

One of the major challenges in IA is the design of precoding and decoding matrices, which has significant impact on the levels of interference leakage and sum rate that IA can achieve. The closed-form solutions to IA were provided in [4] for the MIMO interference channel with three users, one DoF, two transmitting and receiving antennas, and were extended to a more general situation of *N* + 1 users and *N* antennas in [21]. Unfortunately, the problem of designing the optimal precoding and decoding matrices has been proved to be NP-hard [22], and the number of solutions increases rapidly with the scale of the system. Therefore, numerical methods have to be employed to search for the suboptimal solutions. Gomadam *et al.* proposed a minimizing interference leakage (MinIL) algorithm in [23], which highlights the advantage of relatively low signaling overhead by utilizing the channel reciprocity. However, the MinIL algorithm might degrade significantly in the low SNR regime for the reason that it only focuses on suppressing the interference from the unintended users, without considering the power loss of the desired signal. Hence, Gomadam *et al.* modified the method and progressed to the maximizing signal-to-interference-plus-noise (Max-SINR) algorithm in [24], which can improve the throughput especially when the SNR is low. A low-complexity modified alternating minimization algorithm was proposed in [25], which leverages the courant penalty function to jointly optimize the interference leakage and intended signal. The concept of signal leakage was introduced by Shrestha *et al.* in [26], and the IA solutions were searched under the maximal signal-to-leakage-plus-noise ratio criterion. A sequential antenna switching IA algorithm was proposed in [27], where the QoS is improved by selecting proper antenna configurations.

Another challenge that IA confronts is the acquisition of channel state information (CSI), and a large number of works [28–33] have focused on this problem. Sungyoon *et al.* proposed feedback topologies in [29], which can reduce the signaling overhead significantly. An analogy CSI feedback strategy was proposed in [30], which can achieve lower feedback burden while maintaining the full multiplexing gain in the case of comparable forward and backward SNR levels. El Ayach *et al.* designed a CSI training and feedback scheme to maximize the effective sum rate of the IA networks in [32]. Zhao *et al.* have concentrated on the CSI feedback latency in [33] and proposed a new IA scheme by taking advantage of the channel prediction.

## 3. System Model

**Figure 1.**
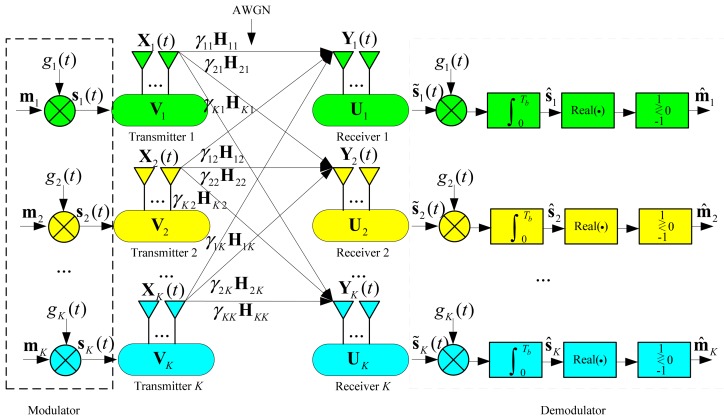
The BPSK transmission system in the *K*-user MIMO interference channel.

In this paper, we concentrate on the BPSK transmission system in the (*M* × *N*, *d*)*^K^* MIMO interference channel as shown in Figure 1. Admittedly, many other types of modulation schemes such as quadrature phase shift keying (QPSK), offset QPSK (O-QPSK) and minimum phase shift keying (MSK) can be applied to the system.

The discussion on the selection of modulation schemes is beyond the scope of this paper, and the readers can refer to [34] for more information. Besides, perfect carrier recovery and symbol synchronization are assumed to be achieved. Therefore, the bandpass waveforms can be transformed to the equivalent baseband signals [35]. As shown in Figure 1, the binary bit vector **m***_k_* ε R*^d^*^×1^ of the *k*-th user is first modulated by the waveform function *g_k_*(*t*), and the modulated waveform vector **s***_k_*(*t*) ε R*^d^*^×1^ can be expressed as:

(1) $$s_{k}(t)=m_{k}g_{k}(t),0\leq t\leq T_{b},k=1,2,\ldots ,K$$

where *T_b_* represents the bit period and:

(2) $$m_{k}={\left[m_{k,1},m_{k,2},\ldots ,m_{k,d}\right]}^{\text{T}},m_{k,l}\in \{+1,-1\},l=1,2,\ldots ,d$$

(3) $$s_{k}(t)={\left[s_{k,1}(t),s_{k,2}(t),\ldots ,s_{k,d}(t)\right]}^{\text{T}}$$

Then the modulated waveform vector is precoded by the precoding matrix **V***_k_* ε C*^M^*^×^*^d^* to produce the transmitted waveform vector, *i.e.*,

(4) $$X_{k}(t)=V_{k}s_{k}(t)$$

where:

(5) $$V_{k}=[V_{k,1},V_{k,2},\ldots ,V_{k,d}]$$

(6) $$V_{k,l}\epsilon {\text{C}}^{M\times 1},l=1,2,\ldots ,d$$

(7) $$\|V_{k,l}\|=1$$

The precoded waveforms will be sent across the interference channel, and the received waveform vector **Y***_k_*(*t*) ε C*^N^*^×1^ at the *k*-th receiver can be expressed as:

(8) $$\begin{array}{ll} Y_{k}(t) & =\overset{K}{\underset{i=1}{\text{∑}}}\gamma_{ki}H_{ki}X_{i}(t)+n_{k}(t)\\ & =\overset{K}{\underset{i=1}{\text{∑}}}\gamma_{ki}H_{ki}V_{i}s_{i}(t)+n_{k}(t),0\leq t\leq T_{b} \end{array}$$

where **n***_k_*(*t*) ε C*^N^*^×1^ denotes the received noise vector at the *k*-th receiver, each element of which is an independent zero-mean additive white Gaussian noise (AWGN) process with two-sided power spectral density of *N*_0_/2 W/Hz; **H***_ki_* ε C*^N^*^×^*^M^* represents the small scale fading channel gain matrix from the *i*-th transmitter to the *k*-th receiver, each entity of which is an independent random variable with the distribution of CN(0,1); γ*_ki_* denotes the large-scale channel attenuation between the *i*-th transmitter and the *k*-th receiver.

Most of the previous works have focused on the symmetric networks, where the users are located in the proximity of each other and share the same path-loss, *i.e.*, γ*_ki_* = 1 for all users. However, in practical communication systems, the users might scatter around geographically. Therefore, we will also consider the asymmetric networks, where the large-scale fading is associated with the distance and is given by [13]:

(9) $$\gamma_{ki}=\sqrt{r_{ki}^{-\alpha }}$$

The notations α and γ*_ki_* represent the path-loss exponent and the distance between the transmitter *i* and receiver *k*, respectively.

At the *k*-th receiver, a decoding matrix **U***_k_* ε C*^N^*^×^*^d^* is applied to suppress the interference from the unintended users, and the reconstructed signal waveform vector can be formulated as:

(10) $$\begin{array}{ll} {\tilde{s}}_{k}(t) & =U_{k}^{\text{H}}Y_{k}(t)\\ & =\overset{K}{\underset{i=1}{\text{∑}}}\gamma_{ki}U_{k}^{\text{H}}H_{ki}V_{i}s_{i}(t)+U_{k}^{\text{H}}n_{k}(t)\\ & =\gamma_{kk}U_{k}^{\text{H}}H_{kk}V_{k}s_{k}(t)+\overset{K}{\underset{i=1,i\neq k}{\text{∑}}}\gamma_{ki}U_{k}^{\text{H}}H_{ki}V_{i}s_{i}(t)+U_{k}^{\text{H}}n_{k}(t) \end{array}$$

where:

(11) $$U_{k}=[U_{k,1},U_{k,2},\ldots ,U_{k,d}]$$

(12) $$U_{k,l}\epsilon {\text{C}}^{N\times 1},l=1,2,\ldots ,d$$

(13) $$\|U_{k,l}\|=1$$

At the demodulator, the received signal vector **s̃**_k_(*t*) is correlated with the match filter to yield the test statistic vector, *i.e.*,

(14) $${\hat{s}}_{k}=\int_{0}^{T_{b}}{\tilde{s}}_{k}(t)g_{k}(t)dt$$

Finally, the demodulated bit vector **m̂***_k_* is determined according to the real part of the test statistic vector, *i.e.*,

(15) $${\hat{m}}_{k,l}=\left\{ \begin{array}{l} 1,\text{when real}({\hat{s}}_{k,l})>0\\ -1,\text{when real}({\hat{s}}_{k,l})<0 \end{array}\right.,l=1,2,\ldots ,d$$

where:

(16) $${\hat{s}}_{k}={\left[{\hat{s}}_{k,1},{\hat{s}}_{k,2},\ldots ,{\hat{s}}_{k,d}\right]}^{\text{T}},{\hat{m}}_{k}={\left[{\hat{m}}_{k,1},{\hat{m}}_{k,2},\ldots ,{\hat{m}}_{k,d}\right]}^{\text{T}}$$

According to [11], the IA feasible condition can be given as follows:

(17) K≤(M+N)/d−1

Under the constraint of Equation (17), the system is likely to be feasible, and perfect IA can be achieved if the following conditions are satisfied:

(18) $$U_{k}^{\text{H}}H_{kj}V_{j}=0;k,j=1,2,\ldots ,K;k\neq j$$

(19) $$\text{rank}(U_{k}^{\text{H}}H_{kk}V_{k})=d$$

Many works have concentrated the design of the precoding and decoding matrices for IA [23–26]. One of the most representative methods is the Max-SINR algorithm as shown in Algorithm 1. The Max-SINR algorithm highlights the advantage of good sum rate achievement. In this paper, the Max-SINR algorithm is selected as the strategy for designing the corresponding matrices. Perfect CSI and large-scale channel attenuation γ*_ki_* are assumed to be known at each node. Although the situations of imperfect CSI are interesting and more practical, detailed discussion on CSI feedback is beyond the scope of this paper. The readers who are interested in this area can refer to the “Related Work” section and the references herein. Besides, we assume that the nodes in the WSNs can communicate with each other. In the scenario where the distances between some nodes are too large, the relay nodes [36,37] can be added to transmit data between them.


**Algorithm 1** Max-SINR Algorithm

Set the maximal iterations and the initial precoding matrices as **V***_k_* (*k* = 1, 2,…, *K*). The columns of **V***_k_* are linearly independent unit vectors.(Forward Iteration) Calculate the interference-plus-noise covariance matrices **J***_k,l_* (*l* = 1, 2, …, *d*) in the original channel, *i.e.*,
(20)$J_{k,l}=\overset{K}{\underset{j=1,j\neq k}{\text{∑}}}H_{kj}V_{j}V_{j}^{\text{H}}H_{kj}^{H}+\overset{d}{\underset{r=1,r\neq l}{\text{∑}}}H_{kk}V_{k,r}V_{k,r}^{\text{H}}H_{kk}^{H}+\sigma^{2}I$Formulate the decoding matrix **U***_k_*, the *l*-th column of which can be represented as
(21)$U_{k,l}=J_{k,l}^{-1}H_{kk}V_{k,l}/\|J_{k,l}^{-1}H_{kk}V_{k,l}\|$(Backward Iteration) Calculate the interference-plus-noise covariance matrices **J⃖***_k,l_* in the reciprocal channel as:
${\overset{\leftarrow }{J}}_{k,l}=\overset{K}{\underset{j=1,j\neq k}{\text{∑}}}H_{jk}^{\text{H}}U_{j}U_{j}^{\text{H}}H_{jk}+\overset{d}{\underset{r=1,r\neq l}{\text{∑}}}H_{kk}^{\text{H}}U_{k,r}U_{k,r}^{\text{H}}H_{kk}+\sigma^{2}I$Formulate the decoding matrix **V***_k_*, the *l*-th column of which can be represented as
$V_{k,l}={\overset{\leftarrow }{J}}_{k,l}^{-1}H_{kk}^{\text{H}}U_{k,l}/\|{\overset{\leftarrow }{J}}_{k,l}^{-1}H_{kk}^{\text{H}}U_{k,l}\|$Repeat step (2) and (3) until the algorithm converges or the maximal iterations number is reached.


## 4. The Joint Spatial-Code Clustered IA Scheme

The conventional IA can achieve low interference leakage and high throughput when the number of users satisfies the IA feasibility condition. However, in large-scale networks with limited antennas, the IA feasibility condition cannot be met, and the interference leakage may become intense, giving rise to significant degradation of the QoS. Since the interference from the unintended users cannot be eliminated only through the spatial domain, extra dimensions have to be introduced to address this problem. In this section, we jointly explore the spatial as well as the code dimensions, and propose a novel joint spatial-code clustered IA scheme to mitigate the interference that the conventional IA cannot suppress effectively. Moreover, most of the existing works mainly concentrate on IA from the perspectives of sum rate and DoFs, without further analyzing the error performance. However, in the practical communication systems, BER serves as an important measurement of the QoS. Therefore, we formulate the BER expressions of the proposed JSCC-IA scheme in the cases of identical and different propagation delays, respectively.

### 4.1. The Mechanism of the JSCC-IA Scheme

**Figure 2.**
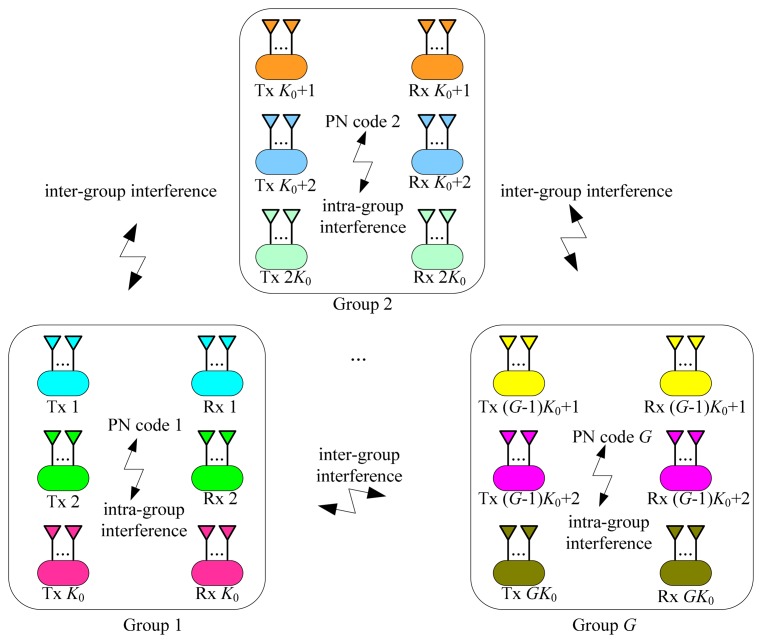
The JSCC-IA scheme with *G* groups and *K*_0_ users in each group.

The JSCC-IA scheme is depicted in Figure 2 for the (*M* × *N*, *d*)*^K^* interference channel where the number of users exceeds the IA feasibility constraint, *i.e.*, *K* > (*M* + *N*)/*d* − 1. The basic idea of the JSCC-IA scheme is to partition the users into *G* groups, with *K*_0_ users in each group that satisfies:

(22) $$K_{0}\leq (M+N)/d-1$$

Therefore, the precoding and decoding matrices can be designed to mitigate the intra-group interference just as the conventional IA. The key point of the JSCC-IA scheme is that the data within each group is modulated by a distinct PN code to suppress the inter-group interference according to the idea of spread spectrum. Therefore, the inter-group and intra-group interference can be respectively aligned in the code and spatial domains, and thus the networks can accommodate many more users to communicate simultaneously via the same frequency band.

Without loss of generality, we assign the *k*-th user to the ⌈*k/K*_0_⌉-th group, and the *j*-th group is assigned with the PN sequence *C_j_*(*n*). We will employ *k* to represent ⌈*k/K*_0_⌉ henceforth to avoid cumbersome notation. The PN sequence *C_j_*(*n*) can be given by:

(23) $$C_{j}(n)=\left\{ \begin{array}{l} \pm 1,0\leq n\leq L-1\\ 0,\text{else} \end{array}\right.$$

where *L* is the length of the PN sequence. Then the baseband waveform associated with *C_j_*(*n*) can be expressed as:

(24) $$c_{j}(t)=A\overset{L-1}{\underset{n=0}{\text{∑}}}C_{j}(n){\text{rect}}_{T_{c}}(t-nT_{c}),0\leq t\leq LT_{c}$$

where *A* and *T_c_* represent the amplitude of the transmitted signal and the duration of one PN chip, respectively. The relationship of *T_b_*, *T_c_* and *L* can be expressed as:

(25) $$T_{b}=LT_{c}$$

Therefore, the waveform function for the JSCC-IA system can be given by:

(26) $$g_{k}(t)=c_{\underline{k}}(t)$$

And the bit energy *E_b_* of the JSCC-IA scheme can be calculated as:

(27) $$E_{b}=\int_{0}^{T_{b}}{\left[m_{k,l}g_{k}(t)\right]}^{2}dt=\int_{0}^{T_{b}}{\left[g_{k}(t)\right]}^{2}dt=\int_{0}^{T_{b}}{\left[c_{\underline{k}}(t)\right]}^{2}dt=A^{2}T_{b}=LA^{2}T_{c}$$

The most significant difference between the proposed JSCC-IA and the conventional IA systems lies in the receiver, and the demodulator of the JSCC-IA scheme can suppress the inter-group interference by correlating the received signal with the local PN waveform. To facilitate the analysis in the next subsections, we define the correlation between two PN sequences as:

(28) $$\rho_{ij}(l)=\overset{L-1}{\underset{n=0}{\text{∑}}}C_{i}(n)C_{j}(n-l)$$

According to the properties of PN sequences, the autocorrelation value ρ*_ii_*(0), which equals to *L*, is significantly larger than the cross-correlation value ρ*_ij_*(0) with *i* ≠ *j*. Therefore, the correlation of the PN codes can not only provide extra gains to the desired signals within each cluster, but also mitigate the interference from the unintended groups. There are various types of PN sequences such as m-sequence, M-sequence and Gold sequence. We employ the Gold sequence in the JSCC-IA system due to its advantage of low cross-correlation value, easy implementation, large number of available sequences and so on. Further discussion on the properties of different PN sequences is beyond the scope of this paper, and the readers can refer to [34] for more details.

### 4.2. Error Performance of the JSCC-IA Scheme with Identical Propagation Delay

In the JSCC-IA system, when all users share the same propagation delay, the reconstructed signal waveform vector at the *k*th receiver can be expressed as Equation (10). The demodulator employs *g_k_*(*t*) to correlate with **s̃***_k_*(*t*), and the test statistic vector can be written as:

(29) $${\hat{s}}_{k}=\int_{0}^{T_{b}}\overset{K}{\underset{i=1}{\text{∑}}}\gamma_{ki}U_{k}^{\text{H}}H_{ki}V_{i}m_{i}g_{i}(t)g_{k}(t)dt+\int_{0}^{T_{b}}U_{k}^{\text{H}}n_{k}(t)g_{k}(t)dt$$

From Equations (24) and (26)–(28), the correlation between *g_i_*(*t*) and *g_k_*(*t*) can be calculated as:

(30) $$\begin{array}{ll} \int_{0}^{T_{b}}g_{i}(t)g_{k}(t)dt & =\int_{0}^{T_{b}}c_{\underline{i}}(t)c_{\underline{k}}(t)dt\\ & =A^{2}\overset{L-1}{\underset{n=0}{\text{∑}}}\left[C_{\underline{i}}(n)C_{\underline{k}}(n)\int_{nT_{c}}^{(n+1)T_{c}}{\text{rect}}_{T_{c}}(t-nT_{c}){\text{rect}}_{T_{c}}(t-nT_{c})dt\right]\\ & =A^{2}T_{c}\overset{L-1}{\underset{n=0}{\text{∑}}}C_{\underline{k}}(n)C_{\underline{i}}(n)\\ & =E_{b}\rho_{\underline{k}\underline{i}}(0)/L \end{array}$$

Substitute Equation (30) into Equation (29), and the test statistic vector can be rewritten as:

(31) $$\begin{array}{ll} {\hat{s}}_{k} & =\frac{1}{L}\overset{K}{\underset{i=1}{\text{∑}}}E_{b}\gamma_{ki}\rho_{\underline{k}\underline{i}}(0)U_{k}^{\text{H}}H_{ki}V_{i}m_{i}+{\hat{n}}_{k}\\ & =\frac{1}{L}E_{b}\gamma_{kk}\rho_{\underline{k}\underline{k}}(0)U_{k}^{\text{H}}H_{kk}V_{k}m_{k}+\frac{1}{L}\overset{K}{\underset{i=1,i\neq k}{\text{∑}}}E_{b}\gamma_{ki}\rho_{\underline{k}\underline{i}}(0)U_{k}^{\text{H}}H_{ki}V_{i}m_{i}+{\hat{n}}_{k} \end{array}$$

where:

(32) $${\hat{n}}_{k}=\int_{0}^{T_{b}}U_{k}^{\text{H}}n_{k}(t)g_{k}(t)dt$$

represents the statistic noise vector with the property as follows:

#### Theorem 1

*The statistic noise vector at the k-th receiver, i.e.*, **n̂***_k_* = [*n̂_k,1_, n̂_k,2_*, …,*n̂_k,N_*]*^T^* ε *C^N^*^×^*^1^ is a zero-mean Gaussian random vector. Particularly, the variances of n̂_k,l_ and real(n̂_k,l_) can be respectively given by var*[*n̂_k,l_*] = *N_0_E_b_ / 2 and var*[*real(n̂_k,l_)*] = *N_0_E_b_ / 4, with l* = *1, 2*, …,* N*.

#### Theorem 1 of Proof

See Appendix.

As shown in Equation (31), the distortion of **m***_k_* comes from the residual interference and the statistic noise vector **n̂***_k_*, and the received SINR of the *l*-th data stream of the *k*-th user can be calculated as:

(33) $$\begin{array}{ll} {\text{SINR}}_{\text{JSCC}1}(k,l) & =\frac{{\left|E_{b}\gamma_{kk}\rho_{\underline{k}\underline{k}}(0)U_{k,l}^{\text{H}}H_{kk}V_{k,l}m_{k,l}/L\right|}^{2}}{\overset{K}{\underset{i=1,i\neq k}{\text{∑}}}\overset{d}{\underset{j=1}{\text{∑}}}{\left|E_{b}\gamma_{ki}\rho_{\underline{k}\underline{i}}(0)U_{k,l}^{\text{H}}H_{ki}V_{i,j}m_{i,j}/L\right|}^{2}+\overset{d}{\underset{j=1,j\neq l}{\text{∑}}}{\left|E_{b}\gamma_{kk}\rho_{\underline{k}\underline{k}}(0)U_{k,l}^{\text{H}}H_{kk}V_{k,j}m_{k,j}/L\right|}^{2}+N_{0}E_{b}/2}\\ & =\frac{{\left|\gamma_{kk}\rho_{\underline{k}\underline{k}}(0)U_{k,l}^{\text{H}}H_{kk}V_{k,l}\right|}^{2}}{\overset{K}{\underset{i=1,i\neq k}{\text{∑}}}\overset{d}{\underset{j=1}{\text{∑}}}{\left|\gamma_{ki}\rho_{\underline{k}\underline{i}}(0)U_{k,l}^{\text{H}}H_{ki}V_{i,j}\right|}^{2}+\overset{d}{\underset{j=1,j\neq l}{\text{∑}}}{\left|E_{b}\gamma_{kk}\rho_{\underline{k}\underline{k}}(0)U_{k,l}^{\text{H}}H_{kk}V_{k,j}\right|}^{2}+L^{2}N_{0}/2E_{b}} \end{array}$$

Since ρ*_kk_* (0) ≫ ρ*_ki_*(0) when *k* ≠ *i*, the correlation of the PN code can not only increase the desired signal power, but can also suppress the interference from the other groups.

As shown in Equation (31), when **m***_i_* (*i* = 1, 2, …, *K*) is transmitted, the test statistic vector **ŝ***_k_* is a Gaussian random vector with the expected value as:

(34) $$E[{\hat{s}}_{k}]=\frac{1}{L}\overset{K}{\underset{i=1}{\text{∑}}}E_{b}\gamma_{ki}\rho_{\underline{k}\underline{i}}(0)U_{k}^{\text{H}}H_{ki}V_{i}m_{i}$$

Define:

(35) $$F_{ki}=\gamma_{ki}\rho_{\underline{k}\underline{i}}(0)U_{k}^{H}H_{ki}V_{k}\epsilon {\text{C}}^{d\times d}$$

and **E**[**ŝ***_k_*] can be rewritten as:

(36) $$E[{\hat{s}}_{k}]=\frac{E_{b}}{L}\overset{K}{\underset{i=1}{\text{∑}}}F_{ki}m_{i}$$

To attain the expected value of **ŝ***_k_*, all the possible transmitted messages have to be considered, and we introduce the transformation matrix **T**_JSCC1_, the overall bit vector **M** and the overall test statistic vector **s̄** as follows:

(37) $$T_{\text{JSCC}1}=\left[\begin{array}{l} F_{11},F_{12},\ldots ,F_{1K}\\ F_{21},F_{22},\ldots ,F_{2K}\\ \ldots \\ F_{K1},F_{K2},\ldots ,F_{KK} \end{array}\right]\epsilon {\text{C}}^{Kd\times Kd},M=\left[\begin{array}{l} m_{1}\\ m_{2}\\ \ldots \\ m_{K} \end{array}\right]\epsilon {\text{R}}^{Kd\times 1},\bar{s}=\left[\begin{array}{l} {\hat{s}}_{1}\\ {\hat{s}}_{2}\\ \ldots \\ {\hat{s}}_{K} \end{array}\right]\epsilon {\text{C}}^{Kd\times 1}$$

It is obvious that there are 2*^Kd^* possible value of **M**, and we index them as **M**_1_,**M**_2_,…**M**_2_*_Kd_*. From Equations (36) and (37), when **M***_j_* (*j* = 1, 2, …, 2*^Kd^*) is transmitted, the expected value of **s̄** can be calculated as:

(38) $$E[\bar{s}|M_{j}]=(E_{b}/L)T_{\text{JSCC}1}M_{j}$$

Therefore, the expected values of **s̄** under all the transmitted bit vectors can be written as:

(39) $$\begin{array}{cc} \left[E[\bar{s}|M_{1}],E[\bar{s}|M_{2}],\ldots ,E[\bar{s}|M_{2^{Kd}}]\right] & =(E_{b}/L)T_{\text{JSCC}1}\left[M_{1},M_{2},\ldots ,M_{2^{Kd}}\right]\\ & =(E_{b}/L)T_{\text{JSCC}1}{\bar{M}}_{\text{JSCC}1}\\ & =(E_{b}/L)D_{\text{JSCC}1} \end{array}$$

where:

(40) $${\bar{M}}_{\text{JSCC}1}=\left[M_{1},M_{2},\ldots ,M_{2^{Kd}}\right]$$

(41) $$D_{\text{JSCC}1}=T_{\text{JSCC}1}{\bar{M}}_{\text{JSCC}1}$$

Based on the analysis above, the BER expression of the proposed JSCC-IA with identical propagation delay is straightforward and can be expressed as the following theorem.

#### Theorem 2

*The BER of the l-th data stream of the k-th user in the JSCC-IA system with identical propagation delay can be expressed as:*

(42) $${\text{BER}}_{\text{JSCC}1}(k,l)=\frac{1}{2^{Kd}}\overset{2^{Kd}}{\underset{q=1}{\text{∑}}}\left\{\frac{1}{2}+\frac{1}{2}\text{erf}\left[-{\bar{M}}_{\text{JSCC}1}((k-1)d+l,q)\text{real}\left[D_{\text{JSCC}1}((k-1)d+l,q)\right]\frac{1}{L}\sqrt{\frac{2E_{b}}{N_{0}}}\right]\right\}$$

*And the overall BER of the whole system is given by:*

(43) $${\bar{\text{BER}}}_{\text{JSCC}1}=\frac{1}{Kd}\overset{K}{\underset{k=1}{\text{∑}}}\overset{d}{\underset{l=1}{\text{∑}}}{\text{BER}}_{\text{JSCC}1}(k,l)$$

#### Theorem 2 of Proof

For the case where the priori probabilities of the transmitted data are equal, the probability of the transmitted bit vector can be expressed as:

(44) $$P(M_{q})=1/2^{Kd},q=1,2,\ldots ,2^{Kd}$$

As shown in Equation (39), when **M***_q_* is transmitted, the expected value of **s̄** can be given by:

(45) $$E[\bar{s}|M_{q}]=(E_{b}/L)D_{\text{JSCC}1,q}$$

where **D**_JSCC1_,*_q_* represents the *q*-th column of **D**_JSCC1_. As we make the decision according to the real part of the test statistic, the expected value and variance of real(*ŝ_k,l_*) when **M***_q_* is transmitted can be respectively given as:

(46) $$E[\text{real}({\hat{s}}_{k,l})|M_{q}]=(E_{b}/L)\text{real}\left[D_{\text{JSCC}1}((k-1)d+l,q)\right]$$

(47) $$\mathrm{var}[\text{real}({\hat{s}}_{k,l})|M_{q}]=\sigma_{R}^{2}=N_{0}E_{b}/4$$

Therefore, the probability density function of real(*ŝ_k,l_*) can be formulated as:

(48) $$\begin{array}{ll} P(z|M_{q}) & =\frac{1}{\sqrt{2\pi \sigma_{R}^{2}}}\exp\left\{-\frac{{\left\{z-E[\text{real}({\hat{s}}_{k,l})|M_{q}]\right\}}^{2}}{2\sigma_{R}^{2}}\right\}\\ & =\frac{1}{\sqrt{2\pi \sigma_{R}^{2}}}\exp\left\{-\frac{{\left\{z-(E_{b}/L)\text{real}\left[D_{\text{JSCC}1}((k-1)d+l,q)\right]\right\}}^{2}}{2\sigma_{R}^{2}}\right\} \end{array}$$

When *m_k_*,*_l_* = 1, the probability of the error occurrence is:

(49) $$\begin{array}{ll} P(e|M_{q},m_{k,l}=1) & =P(z<0|M_{q},m_{k,l}=1)\\ & =\int_{-\infty }^{0}P(z|M_{q})dz\\ & =\frac{1}{2}+\frac{1}{2}\text{erf}\left\{\frac{-(E_{b}/L)\text{real}\left[D_{\text{JSCC}1}((k-1)d+l,q)\right]}{\sqrt{2\sigma_{R}^{2}}}\right\}\\ & =\frac{1}{2}+\frac{1}{2}\text{erf}\left\{\frac{-m_{k,l}(E_{b}/L)\text{real}\left[D_{\text{JSCC}1}((k-1)d+l,q)\right]}{\sqrt{2\sigma_{R}^{2}}}\right\} \end{array}$$

When *m_k_*,*_l_* = −1, the probability of the error occurrence is:

(50) $$\begin{array}{ll} P(e|M_{q},m_{k,l}=-1) & =P(z>0|M_{q},m_{k,l}=-1)\\ & =\int_{0}^{+\infty }P(z|M_{q})dz\\ & =\frac{1}{2}+\frac{1}{2}\text{erf}\left\{\frac{(E_{b}/L)\text{real}\left[D_{\text{JSCC}1}((k-1)d+l,q)\right]}{\sqrt{2\sigma_{R}^{2}}}\right\}\\ & =\frac{1}{2}+\frac{1}{2}\text{erf}\left\{\frac{-m_{k,l}(E_{b}/L)\text{real}\left[D_{\text{JSCC}1}((k-1)d+l,q)\right]}{\sqrt{2\sigma_{R}^{2}}}\right\} \end{array}$$

As *m_k,l_*= **M̂**_JSCCI_((*k* −1)*d* + *l,q*), we substitute it into Equations (49) and (50) to attain the error probability of *m_k_*,*_l_*, *i.e.*,

(51) $$P\left(e|M_{q}\right)=\frac{1}{2}+\frac{1}{2}\text{erf}\left\{\frac{-(E_{b}/L){\bar{M}}_{\text{JSCC}1}((k-1)d+l,q)\text{real}\left[D_{\text{JSCC}1}((k-1)d+l,q)\right]}{\sqrt{2\sigma_{R}^{2}}}\right\}$$

Substitute Equation (47) into Equation (51), and the error probability of *m_k_*,*_l_* can be expressed as:

(52) $$P\left(e|M_{q}\right)=\frac{1}{2}+\frac{1}{2}\text{erf}\left\{-{\bar{M}}_{\text{JSCC}1}((k-1)d+l,q)\text{real}\left[D_{\text{JSCC}1}((k-1)d+l,q)\right]\frac{1}{L}\sqrt{\frac{2E_{b}}{N_{0}}}\right\}$$

When all the possible transmitted bit vectors are considered, the overall averaged BER of *m_k_*,*_l_* can be formulated as:

(53) $${\text{BER}}_{\text{JSCC}1}(k,l)=P(M_{q})\overset{2^{Kd}}{\underset{q=1}{\text{∑}}}P(e|M_{q})$$

Substitute Equations (44) and (52) into Equation (53), and Equation (42) can be verified.

### 4.3. Error Performance of the JSCC-IA Scheme with Different Propagation Delays

**Figure 3.**
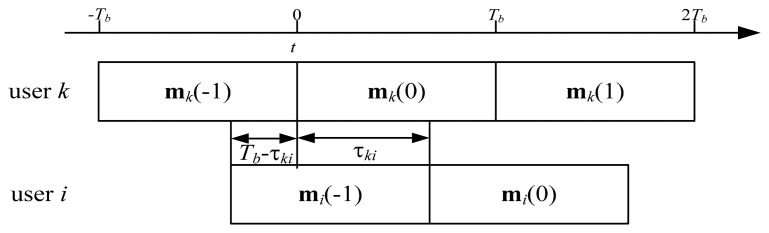
The propagation delay between two users.

Based on the analysis in Section 4.2, we progress to a more complex but practical situation where the users have different propagation delays. As shown in Figure 3, there might be two incomplete message frames during one message interval. Without loss of generality, the propagation delay between the *k*-th and *i*-th users is assumed to be the multiples of the duration of one PN chip, *i.e.*,

(54) $$\tau_{ki}=\Delta_{ki}T_{c},\Delta_{ki}=0,1,\ldots ,L-1$$

Then the reconstructed signal waveform vector at the *k*-th receiver can be expressed as:

(55) $$\begin{array}{ll} {\tilde{s}}_{k}(t) & =\gamma_{kk}U_{k}^{\text{H}}H_{kk}V_{k}m_{k}(0)g_{k}(t)\\ & +\overset{K}{\underset{i=1,i\neq k}{\text{∑}}}\left\{\gamma_{ki}U_{k}^{\text{H}}H_{ki}V_{i}[m_{i}(0)g_{i}(t-\tau_{ki})+m_{i}(-1)g_{i}(t+T_{b}-\tau_{ki})\right]\}\\ & +U_{k}^{\text{H}}n_{k}(t),0\leq t\leq T_{b} \end{array}$$

The demodulator employ *g_k_*(*t*) to correlate with **s̃***_k_*(*t*), and the test statistic vector can be written as:

(56) $$\begin{array}{ll} {\hat{s}}_{k} & =\int_{0}^{T_{b}}{\tilde{s}}_{k}(t)g_{k}(t)dt\\ & =\gamma_{kk}U_{k}^{\text{H}}H_{kk}V_{k}m_{k}(0)\int_{0}^{T_{b}}g_{k}(t)g_{k}(t)dt\\ & +\overset{K}{\underset{i=1,i\neq k}{\text{∑}}}\left\{\gamma_{ki}U_{k}^{\text{H}}H_{ki}V_{i}\left[m_{i}(0)\int_{0}^{T_{b}}g_{k}(t)g_{i}(t-\tau_{ki})dt+m_{i}(-1)\int_{0}^{T_{b}}g_{k}(t)g_{i}(t+T_{b}-\tau_{ki})dt\right]\right\}\\ & +\int_{0}^{T_{b}}U_{k}^{\text{H}}n_{k}(t)g_{k}(t)dt \end{array}$$

From Equations (24), (26), (28) and (54), the integrals in Equation (56) can be rewritten as:

(57) $$\int_{0}^{T_{b}}g_{k}(t)g_{i}(t-\tau_{ki})dt=E_{b}\rho_{\underline{k}\underline{i}}(\Delta_{ki})/L$$

(58) $$\int_{0}^{T_{b}}g_{k}(t)g_{i}(t+T_{b}-\tau_{ki})dt=E_{b}\rho_{\underline{k}\underline{i}}(\Delta_{ki}-L)/L$$

Substitute Equations (57) and (58) into Equation (56), and the test statistic vector can be rewritten as:

(59) $${\hat{s}}_{k}=E_{b}\gamma_{kk}U_{k}^{\text{H}}H_{kk}V_{k}m_{k}(0)+\frac{E_{b}}{L}\overset{K}{\underset{i=1,i\neq k}{\text{∑}}}\left\{\gamma_{ki}U_{k}^{\text{H}}H_{ki}V_{i}\left[m_{i}(0)\rho_{\underline{k}\underline{i}}(\Delta_{ki})+m_{i}(-1)\rho_{\underline{k}\underline{i}}(\Delta_{ki}-L)\right]\right\}+{\hat{n}}_{k}$$

As shown in Equation (59), the desired signal vector **m***_k_*(0) is degraded by the two consecutive messages from the other users, and the received SINR of the *l*-th data stream of the *k*-th user can be calculated as:

(60) $${\text{SINR}}_{\text{JSCC}2}(k,l)=\frac{P_{\text{JSCC}2}(k,l)}{I_{\text{JSCC}2}(k,l)+N_{0}E_{b}/2}$$

where:

(61) $$P_{\text{JSCC}2}(k,l)={\left|E_{b}\gamma_{kk}\rho_{\underline{k}\underline{k}}(0)U_{k,l}^{\text{H}}H_{kk}V_{k,l}m_{k,l}/L\right|}^{2}$$

(62) $$\begin{array}{ll} I_{\text{JSCC}2}(k,l) & =\overset{K}{\underset{i=1,i\neq k}{\text{∑}}}\overset{d}{\underset{j=1}{\text{∑}}}{\left|E_{b}\gamma_{ki}\rho_{\underline{k}\underline{i}}(\Delta_{ki})U_{k,l}^{\text{H}}H_{ki}V_{i,j}m_{i,j}(0)/L\right|}^{2}\\ & +\overset{K}{\underset{i=1,i\neq k}{\text{∑}}}\overset{d}{\underset{j=1}{\text{∑}}}{\left|E_{b}\gamma_{ki}\rho_{\underline{k}\underline{i}}(\Delta_{ki}-L)U_{k,l}^{\text{H}}H_{ki}V_{i,j}m_{i,j}(-1)/L\right|}^{2}\\ & +\overset{d}{\underset{j=1,j\neq l}{\text{∑}}}{\left|E_{b}\gamma_{kk}\rho_{\underline{k}\underline{k}}(0)U_{k,l}^{\text{H}}H_{kk}V_{k,j}m_{k,j}/L\right|}^{2} \end{array}$$

From Theorem 1, the statistic noise vector **n̂***_k_* is a Gaussian random vector, each component of which is a zero-mean Gaussian variable with variance *N*_0_*E_b_*/2. As shown in Equation (59), the expected value of **ŝ***_k_* is determined by the transmitted bit vectors **m**_1_(−1), **m**_1_(0), …, **m***_k_*_−1_(−1), **m***_k_*_−1_(0), **m***_k_*(0), **m***_k_*_+1_(−1), **m***_k_*_+1_(0), …, **m***_K_*(−1), **m***_K_*(0). Similar as Equation (40), we introduce a (2*K* − 1)*d* × 2^(2^*^K^*^−1)^*^d^* transmitted data matrix **M̄**_JSCDC2_, the column of which represents each possible transmitted data vector. In addition, we also introduce the transformation matrix for the JSCC-IA system with different propagation delays as:

(63) $$T_{\text{JSCC}2}=\left[\begin{array}{l} LQ_{11},\rho_{\underline{1}\underline{2}}(\Delta_{12})Q_{12},\rho_{\underline{1}\underline{2}}(L-\Delta_{12})Q_{12},\ldots ,\rho_{\underline{1}\underline{K}}(\Delta_{1K})Q_{1K},\rho_{\underline{1}\underline{K}}(L-\Delta_{1K})Q_{1K}\\ \rho_{\underline{2}\underline{1}}(\Delta_{21})Q_{21},\rho_{\underline{2}\underline{1}}(L-\Delta_{21})Q_{21},LQ_{22},\ldots ,\rho_{\underline{2}\underline{K}}(\Delta_{2K})Q_{2K},\rho_{\underline{2}\underline{K}}(L-\Delta_{2K})Q_{2K}\\ \ldots \\ \rho_{\underline{K}\underline{1}}(\Delta_{K1})Q_{K1},\rho_{\underline{K}\underline{1}}(L-\Delta_{K1})Q_{K1},\ldots ,\rho_{\underline{K}\underline{K-1}}(\Delta_{K(K-1)})Q_{K(K-1)},\rho_{\underline{K}\underline{K-1}}(L-\Delta_{K(K-1)})Q_{K(K-1)},LQ_{KK} \end{array}\right]$$

where $Q_{ij}=\gamma_{ij}U_{i}^{\text{H}}H_{ij}V_{j}\epsilon {\text{C}}^{d\times d}$. Then the expected values matrix of **s̄** under different transmitted data can be expressed as:

(64) $$(E_{b}/L)T_{\text{JSCC}2}{\bar{M}}_{\text{JSCC}2}=(E_{b}/L)D_{\text{JSCC}2}$$

Then the BER for the JSCC-IA scheme with different propagation delays can be expressed as the follow theorem.

#### Theorem 3

*The BER of the l-th data stream of the k-th user in the JSCC-IA scheme with different propagation delays can be expressed as:*

(65) $${\text{BER}}_{\text{JSCC}2}(k,l)=\frac{1}{2^{(2K-1)d}}\overset{2^{(2K-1)d}}{\underset{q=1}{\text{∑}}}\left\{\frac{1}{2}+\frac{1}{2}\text{erf}\left[-{\bar{M}}_{\text{JSCC}2}(2(k-1)d+l,q)\text{real}\left[D_{\text{JSCC}2}(2(k-1)d+l,q)\right]\frac{1}{L}\sqrt{\frac{2E_{b}}{N_{0}}}\right]\right\}$$

*And the overall averaged BER of the whole system can be expressed as:*

(66) $${\bar{\text{BER}}}_{\text{JSCC}2}=\frac{1}{Kd}\overset{K}{\underset{k=1}{\text{∑}}}\overset{d}{\underset{l=1}{\text{∑}}}{\text{BER}}_{\text{JSCC}2}(k,l)$$

#### Theorem 3 of Proof

The proof for Theorem 3 is similar as that of Theorem 2.

### 4.4. Error Performance of the Conventional IA scheme

To make a comparison between the proposed JSCC-IA and the conventional IA schemes, the error performance of the latter one is investigated in this subsection. The equivalent baseband waveform function and the bit energy *E_b_* of the BPSK signal in the conventional IA system can be respectively expressed as:

(67) $$g_{k}(t)=A\;{\text{rect}}_{T_{b}}(t)$$

(68) $$E_{b}=\int_{0}^{T_{b}}g_{k}(t)g_{k}(t)dt=A^{2}T_{b}$$

Substitute Equations (1), (10), (67) and (68) into Equation (14), and the test statistic vector can be rewritten as:

(69) $${\hat{s}}_{k}=E_{b}\gamma_{kk}U_{k}^{\text{H}}H_{kk}V_{k}m_{k}+\overset{K}{\underset{i=1,i\neq k}{\text{∑}}}E_{b}\gamma_{ki}U_{k}^{\text{H}}H_{ki}V_{i}m_{i}+{\hat{n}}_{k}$$

Therefore, the SINR of the *l*-th data stream of the *k*-th user can be calculated as:

(70) $${\text{SINR}}_{\text{IA}}(k,l)=\frac{{\left|E_{b}\gamma_{kk}U_{k,l}^{\text{H}}H_{kk}V_{k,l}m_{k,l}\right|}^{2}}{\overset{K}{\underset{i=1,i\neq k}{\text{∑}}}\overset{d}{\underset{j=1}{\text{∑}}}{\left|E_{b}\gamma_{ki}U_{k,l}^{\text{H}}H_{ki}V_{i,j}m_{i,j}\right|}^{2}+\overset{d}{\underset{j=1,j\neq l}{\text{∑}}}{\left|E_{b}\gamma_{kk}U_{k,l}^{\text{H}}H_{kk}V_{k,j}m_{k,j}\right|}^{2}+N_{0}E_{b}/2}$$

Similar as the JSCC-IA scheme with identical propagation delay, we introduce the matrix **M̄** as Equation (40) and the the transformation matrix **T**_IA_ as follows:

(71) $$T_{\text{IA}}=\left[\begin{array}{c} B_{11},B_{12},\ldots ,B_{1K}\\ B_{21},B_{22},\ldots ,B_{2K}\\ \ldots \\ B_{K1},B_{K2},\ldots ,B_{KK} \end{array}\right]\epsilon {\text{C}}^{Kd\times Kd}$$

where $B_{ij}=\gamma_{ij}U_{i}^{\text{H}}H_{ij}V_{j}$. Then the BER expression of the conventional IA system can be expressed as the following theorem.

#### Theorem 4

*The BER of the l-th data stream of the k-th user in the conventional IA system can be expressed as:*

(72) $${\text{BER}}_{\text{IA}}(k,l)=\frac{1}{2^{Kd}}\overset{2^{Kd}}{\underset{q=1}{\text{∑}}}\left\{\frac{1}{2}+\frac{1}{2}\text{erf}\left[-\text{sign}[\bar{M}((k-1)d+l,q)]\text{real}\left[D_{\text{IA}}((k-1)d+l,q)\right]\sqrt{\frac{2E_{b}}{N_{0}}}\right]\right\}$$

*And the overall averaged BER of the whole system can be expressed as:*

(73) $${\bar{\text{BER}}}_{\text{IA}}=\frac{1}{Kd}\overset{K}{\underset{k=1}{\text{∑}}}\overset{d}{\underset{l=1}{\text{∑}}}{\text{BER}}_{\text{IA}}(k,l)$$

#### Theorem 4 of Proof

The proof for Theorem 4 is similar as that of Theorem 2.

In this section, we have introduced the JSCC-IA scheme and formulated the associated BER expressions analytically. We have clustered the users randomly, without further considering the effect of different grouping combinations. In the next section, we turn to more practical situations of asymmetric networks, and propose a grouping algorithm to select more appropriate clustering combinations with better performance.

## 5. The Random Grouping Selection Algorithm for the JSCC-IA Scheme in the Asymmetric Networks

The proposed JSCC-IA scheme can improve the BER performance of the IA system in the large-scale networks with limited antennas. However, for asymmetric networks where the attenuation varies significantly among the users, the way how the users are clustered might have an impact on the BER performance, and the simple grouping strategy in Section 4 might not fully explore the potential of the JSCC-IA scheme. In this section, a random grouping selection algorithm is proposed to search for better clustering combinations which can further improve the error performance of the proposed JSCC-IA scheme.

### 5.1. The Random Grouping Selection Algorithm

In the JSCC-IA scheme with *G* groups and *K*_0_ users per group, the number of all possible clustering combinations is $D=C_{GK_{0}}^{K_{0}}C_{(G-1)K_{0}}^{K_{0}}\ldots C_{2K_{0}}^{K_{0}}$ and the set that consists all the possible combinations is S = {*P*_1_, *P*_2_,…, *P_D_*}. Therefore, the optimal combination under the criterion of optimal BER can be expressed as:

(74) $$P_{\text{opt}-\text{BER}}=\arg\underset{P_{m}}{\min}\left\{{\bar{\text{BER}}}_{\text{JSCC}},m=1,2,\ldots ,D\right\}$$

where ${\bar{\text{BER}}}_{\text{JSCC}}$ represents ${\bar{\text{BER}}}_{\text{JSCC}1}$ or ${\bar{\text{BER}}}_{\text{JSCC}2}$ for the JSCC-IA systems with identical or different propagation delays, respectively.

Although the objective function in Equation (74) can achieve the optimal overall error performance, the computational complexity of the theoretical BER by Equation (42) or Equation (65) increases exponentially with the number of users, which hinders the application of the grouping selection strategy. Therefore, we introduce the minimal received SINR as the alternative objective function, *i.e.*,

(75) $$P_{\text{opt}-\text{SINR}}=\arg\underset{P_{m}}{\max}\left\{\underset{k,l}{\min}\left\{{\text{SINR}}_{\text{JSCC}}(k,l),k=1,2,\ldots K,l=1,2,\ldots ,d\right\},m=1,2,\ldots ,D\right\}$$

where SINR_JSCC_ represents SINR_JSCC1_ or SINR_JSC2_ for the JSCC-IA systems with identical or different propagation delays, respectively. Since the received SINR has a significant impact on the error performance, the minimal received SINR can serve as the measurement of the QoS, and the grouping combination that has the highest minimal SINR is chosen as the optimal solution in Equation (75). Moreover, the computational complexity of calculating the received SINR is much lower than that of the BER. Therefore, the proposed objective function can serve as a good measurement to evaluate the performance of different grouping combinations with low computational complexity.

When the number of users is small, brute-force searching (BFS) can be employed to select the optimal grouping combination. However, the number of candidates increases dramatically with the network scale, and searching the optimal clustering combination is a NP-hard problem. Therefore, it is impossible to enumerate all the possible combinations in the case of massive users due to the limited computational time in the practical systems. For the high price of searching for the optimal combination, we can turn to the suboptimal solutions by the RGS algorithm with much lower computational complexity and good performance.

As shown in Algorithm 2, the RGS algorithm provides a grouping combination which can achieve the preset objective minimal received SINR ξ, or returns the one that has the highest minimal SINR within the Θ tested combinations. In the small size networks, Θ can be set as *D* so that all the available candidates can be tested, and the optimal grouping combination can be attained. However, in the large scale networks, *D* is such a huge number that Θ should be much smaller than *D*. Although the solution in the case of Θ < *D* is not optimal, the RGS JSCC-IA scheme can improve the error performance significantly without too much additional computational complexity.


**Algorithm 2** Random Grouping Selection Algorithm

Set the maximal number of iterations as Θ and the objective minimal received SINR as ξ. The variable *i* is used to represent the number of iterations with initial value of 1. Select Θ grouping combinations randomly from S as $S^{\prime }=\{{P^{\prime }}_{1},{P^{\prime }}_{2},\ldots {P^{\prime }}_{\Theta }\}$. Begin iteration.Select the grouping combination ${P^{\prime }}_{i}$ from *S′*, and calculate the precoding and decoding matrices of each group according to the Max-SINR algorithm as W*_i_* = {{**V***_i_*},{**U***_i_*}}.Calculate the SINR of all the data streams according to Equation (33) or Equation (60), and attain the minimal SINR as ξ*_i_*.If *i* = 1, set λ = ξ_1_, *P*_opt_ = *P*_1_ and W_opt_ = W_1_; otherwise, if ξ*_i_* ≥ λ, set λ = ξ*_i_*, *P*_opt_ = *P_i_*, and W_opt_ = W*_i_*.If λ > ξ or *i* > Θ, stop and output *P*_opt_ and W_opt_; otherwise update *i* ← *i* + 1 and go to step (2).


Furthermore, in the case of small ξ, the actual number of iterations is likely to be smaller than Θ, and the complexity of RGS algorithm is low at the expense of little improvement. On the contrary, when ξ is large, the RGS algorithm might have to take much more iterations to search the solution, and the improvement is expected to be significant at the expense of larger computational cost. Therefore, the RGS algorithm provides a good tradeoff between the performance improvement and the computational complexity by adjusting the preset parameters. In addition, a large number of low complexity IA algorithms have recently been developed to attain **V** and **U** with a remarkable fast convergence speed [25,38]. The development of very-large-scale integration (VLSI) also makes it much faster to complete the calculation. Therefore, in the practical situation, Θ can be set to be relatively large, and the possibility that ξ cannot be reached is small.

### 5.2. The Complexity Analysis

In this subsection, the computational complexity of the proposed RGS algorithm is analyzed and compared with the BFS algorithm. The number of complex multiplications is employed as the measurement of the complexity. As shown in Algorithm 2, the computation complexity mainly comes from the Max-SINR algorithm in step (2) and the SINR calculation in step (3). Detail analysis on each step is provided as follows:

(1) From the description of the Max-SINR method in Algorithm 1, for the *k*-th user's interference-plus-noise covariance matrices **J***_k_*,*_l_*, we can firstly calculate the terms **H***_kj_***V***_j_* with *MNd* complex multiplications and then compute the product of **H***_kj_***V***_j_* and (**H***_kj_***V***_j_*)^H^ which takes *N*^2^*d* complex multiplications. Therefore, the number of complex multiplications required by calculating the interference-plus-noise covariance matrix of each data stream can be expressed as (*K*_0_ − 1)(*NMd* + *N*^2^*d*) + (*d* − 1)(*NM* + *N*^2^). As there are *Kd* data streams in total, the sum complexity for calculating all the interference-plus-noise covariance matrices is *Kd*[(*K*_0_ − 1)(*NMd* + *N*^2^*d*) + (*d* − 1)(*NM* + *N*^2^)].
(2) For the decoding vector **U***_k,l_*, the calculation of $J_{k,l}^{-1}$ requires (*N* − 1)!(*N* − 2)*N*^2^ + *N*!(*N* − 1) complex multiplications and one complex division. The multiplication of $J_{k,l}^{-1}$, **H***_kk_* and **V***_k,l_* takes *MN*^2^ + *NM* complex multiplications and the normalization needs *N* complex multiplications and *N* complex divisions. Therefore, it requires (*N* − 1)!(*N* − 2)*N*^2^ + *N*!(*N* − 1) + *MN*^2^ + *NM* + *N* complex multiplications and *N* complex divisions to attain each decoding vector. As there are *Kd* data streams in total, the sum complexity for calculating all the decoding vectors is *Kd*[(*N* − 1)!(*N* − 2)*N*^2^ + *N*!(*N* − 1) + *MN*^2^ + *NM* + *N*] complex multiplications and *KNd* complex divisions.
(3) The complexity for calculating **J⃖***_k,l_* is similar as that of **J***_k,l_*, and the total number of complex multiplications is *Kd*[(*K*_0_ − 1)(*NMd* + *M*^2^*d*) + (*d* − 1)(*NM* + *M*^2^)].
(4) The complexity for calculating **V***_k_* is similar as that of **U***_k_*, and the total number of complex multiplications is *Kd*[(*M* − 1)!(*M* − 2)*M*^2^ + *M*!(*M* − 1)+*NM*^2^ + *NM* + *M*].
(5) For the JSCC-IA scheme with identical propagation delay, the calculation of the SINR for each data stream requires *Kd*[(*MN* + *M*) + 3] complex multiplications and one real division according to Equation (33). For the JSCC-IA scheme with different propagation delays, the calculation of SINR requires (2*K* − 1)*d*[(*MN* + *M*) + 3] complex multiplications and one real division according to Equation (60). As there are *Kd* data streams in total, the overall computational complexity for all the data streams is *K*^2^*d*^2^[(*MN* + *M*) + 3] and *K*(2*K* − 1)*d*^2^[(*MN* + *M*) + 3] for the identical and different propagation delays cases, respectively.

In all, the total numbers of complex multiplications per iteration for the RGS algorithm with the identical and different propagation delays can be respectively summarized as follows:

(76) $$\begin{array}{ll} c_{1} & =Kd[(K_{0}-1)(\text{NMd}+N^{2}d)+(d-1)(NM+N^{2})]+\\ & Kd[(N-1)!(N-2)N^{2}+N!(N-1)+MN^{2}+NM+N]+\\ & Kd[(K_{0}-1)(\text{NMd}+M^{2}d)+(d-1)(NM+M^{2})]+\\ & Kd[(M-1)!(M-2)M^{2}+M!(M-1)+M^{2}N+NM+M]+\\ & K^{2}d^{2}[(MN+M)+3] \end{array}$$

(77) $$\begin{array}{ll} c_{2} & =Kd[(K_{0}-1)(\text{NMd}+N^{2}d)+(d-1)(NM+N^{2})]+\\ & Kd[(N-1)!(N-2)N^{2}+N!(N-1)+MN^{2}+NM+N]+\\ & Kd[(K_{0}-1)(\text{NMd}+M^{2}d)+(d-1)(NM+M^{2})]+\\ & Kd[(M-1)!(M-2)M^{2}+M!(M-1)+M^{2}N+NM+M]+\\ & K(2K-1)d^{2}[(MN+M)+3]. \end{array}$$

The BFS will have to take *D* iterations, and the corresponding overall complexity for the cases of identical and different propagation delays can be respectively represented as:

(78) $$C_{\text{BFS}1}=Dc_{1}$$

(79) $$C_{\text{BFS}2}=Dc_{2}$$

On the contrary, the RGS algorithm only requires Θ iterations at most, and the associated overall computational complexity for the cases of identical and different propagation delays can be respectively represented as:

(80) $$C_{\text{RGS}1}=\Theta c_{1}$$

(81) $$C_{\text{RGS}2}=\Theta c_{2}$$

Table 1 lists the overall complexity of the RGS and BFS algorithms under different number of antennas, users, data streams and groups. The maximal number of iterations for RGS algorithm is chosen as Θ = 20. From the results, we can see that the number of complex multiplications of the BFS algorithm increases dramatically with the network scale, and the complexity is so huge that it is infeasible in the practical systems. On the contrary, the complexity of the RGS algorithm is several orders lower than that of the BFS algorithm, and does not increase rapidly when the network scale becomes larger. From the rate of the complexity between the two algorithms, it can be seen that the proposed RGS algorithm can reduce the computational cost significantly.

## 6. Numerical Results

In this section, we evaluate the proposed JSCC-IA scheme and compare it with the conventional IA system in large-scale networks with limited antennas. The MIMO interference networks (4 × 4, 1)^10^, (4 × 4, 1)^20^ and (6 × 6, 2)^18^ are considered. According to the IA feasibility condition, when four antennas are used, the maximal numbers of users are seven and three for the cases of single and double DoFs, respectively. The JSCC-IA system partitions the users into two and four groups with five users in each cluster for the (4 × 4, 1)^10^ and (4 × 4, 1)^20^ networks, respectively. Similarly, the users in (6 × 6, 2)^18^ are divided into six groups with three users in each cluster. In the first subsection, the symmetric networks are considered, and the error performance, minimal received SINR and average received SINR are evaluated for the proposed and the conventional IA schemes. In the second subsection, we turn to more practical situations of asymmetric networks. The RGS algorithm is discussed in terms of BER and received SINR. In each figure below, the performances are averaged over 200 realizations of randomly generated channel coefficients. The Max-SINR algorithm is chosen to design the precoding and decoding matrices with the iterations of 1000. The Gold sequences are selected as the PN codes with the length of 63 in the following simulations.

### 6.1. Performance in the Symmetric Networks

**Figure 4.**
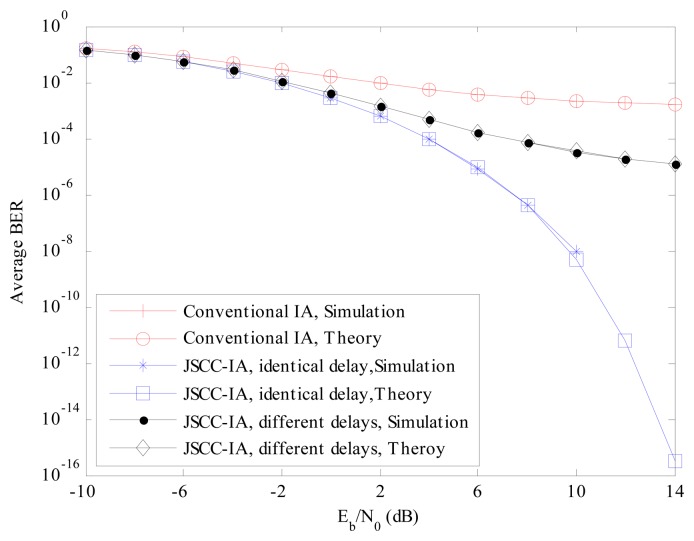
The average theoretical and experimental BER performances of the conventional IA scheme and the JSCC-IA schemes with identical/different propagation delays in the (4 × 4, 1)^10^ symmetric networks.

The theoretical and experimental BER performances of the proposed JSCC-IA schemes with identical and different propagation delays for the (4 × 4, 1)^10^ network are provided and compared with those of the conventional IA scheme in Figure 4.

From the results, it can be seen that the experimental BER agrees very well with the theoretical one, verifying the analytical BER expressions in Section 4. For the JSCC-IA scheme with the same propagation delay, the experimental BER becomes zero when *E_b_*/*N*_0_ is larger than 10 dB due to the finite length of the simulation messages. It is expected that the performance of the traditional IA system is significantly degraded by the strong interference that cannot be mitigated only by the spatial adjustment. The average BER of the conventional IA scheme stays at a very high level, and can only reach the level of 10^−3^ at *E_b_*/*N*_0_ = 14 dB, which is certainly inadequate for the practical requirements. However, when the code domain is introduced, there is a substantial improvement in the error performance. In the JSCC-IA system with identical propagation delay, the BER can be suppressed to the level of 10^−5^ only at *E_b_*/*N*_0_ of 4 dB. The BER of the JSCC-IA scheme decreases rapidly with the raise in *E_b_*/*N*_0_, and the theoretical value can reach the degree of 10^−16^ at *E_b_*/*N*_0_ of 14 dB. For the JSCC-IA system with different propagation delays, the performance might be degraded by the various delays, and the associated BER becomes higher. In spite of this, its performance is still much superior to that of the conventional IA system, with BER of 10^−5^ at *E_b_*/*N*_0_ of 8 dB.

**Figure 5.**
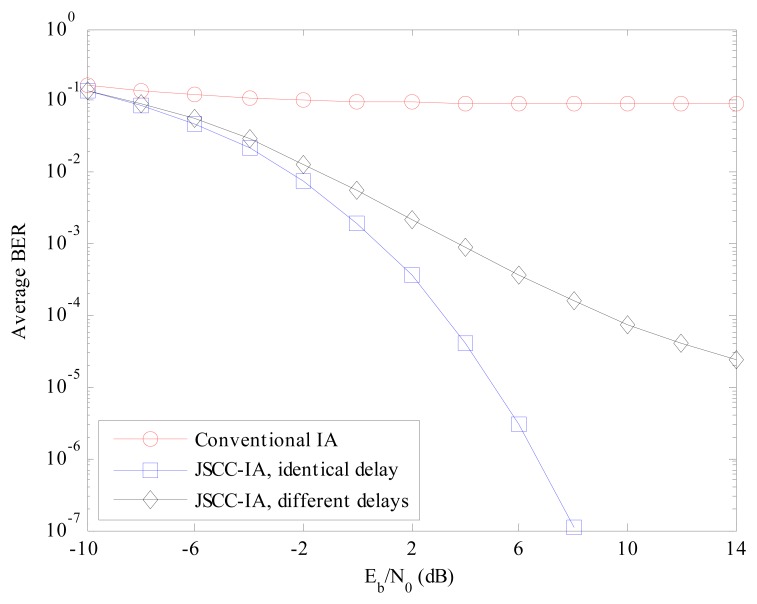
The average experimental BER performances of the conventional IA scheme and the JSCC-IA schemes with identical/different propagation delays in the (4 × 4, 1)^20^ symmetric networks.

To further evaluate the performances in the large-scale WSNs, the scenarios with even more users are considered, and the experimental BER in the (4 × 4, 1)^20^ network is presented in Figure 5. As the number of users is much larger than that the conventional IA scheme can accommodate, the average BER of the conventional IA scheme stays around the high level of 10^−1^. On the contrary, the JSCC-IA scheme can suppress the BER to a much lower level. Considering the *E_b_*/*N*_0_ of 8 dB, the associated BER is 10^−7^ and 10^−4^ for the JSCC-IA schemes with identical and different propagation delays, respectively. When *E_b_*/*N*_0_ increases, the proposed scheme can achieve even better BER.

**Figure 6.**
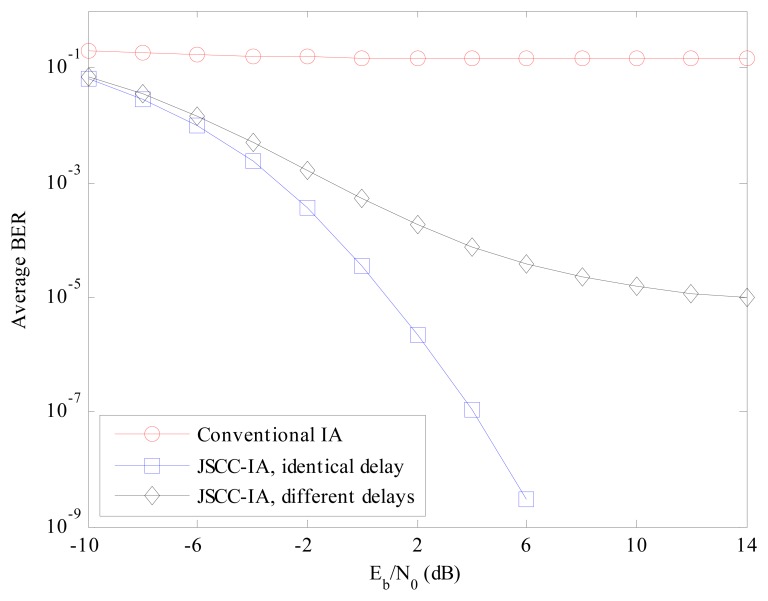
The average experimental BER performances of the conventional IA scheme and the JSCC-IA schemes with identical/different propagation delays in the (6 × 6, 2)^18^ symmetric networks.

Aside from the single DoF circumstance, we progress to a more complicated double data streams situation, and the performances in the (6 × 6, 2)^18^ network are depicted in Figure 6. The BER of the conventional IA system still stays at the unacceptable high value of 10^−1^, and does not show significant improvement with the raise in *E_b_*/*N*_0_. On the other hand, the proposed JSCC-IA scheme exhibits much better BER than the conventional IA system. For the scenario of identical propagation delay, the BER decreases dramatically with the rise in *E_b_*/*N*_0_, and reaches the level of 10^−9^ at *E_b_*/*N*_0_ = 6 dB. Although the performance of the JSCC-IA scheme with different propagation delays is inferior to that with the same delay, it still shows good performance, with BER of 10^−5^ at 4 dB. Therefore, simulations validate the application of JSCC-IA in multi-data-stream networks. Consequently, the proposed JSCC-IA scheme exhibits an implicit advantage over the conventional IA system in term of BER performance.

**Figure 7.**
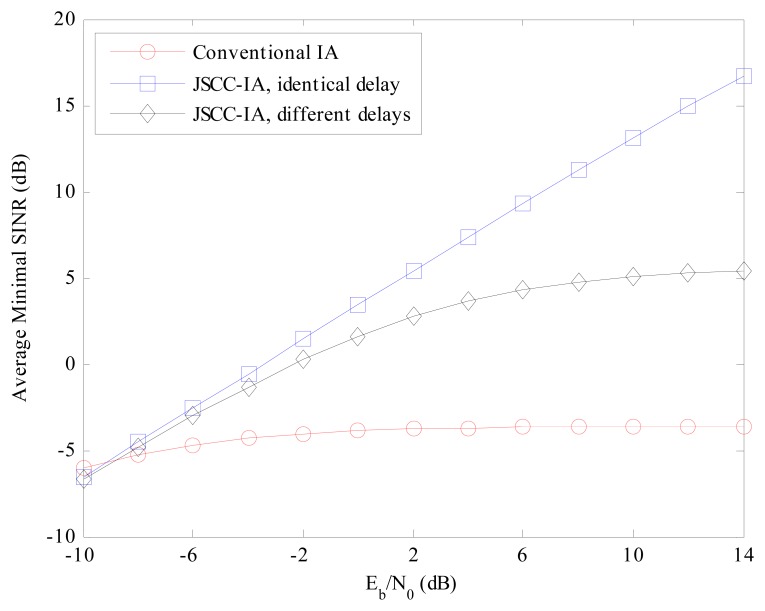
The average minimal received SINR of the conventional IA scheme and the JSCC-IA schemes with identical/different propagation delays in the (4 × 4, 1)^20^ symmetric networks.

**Figure 8.**
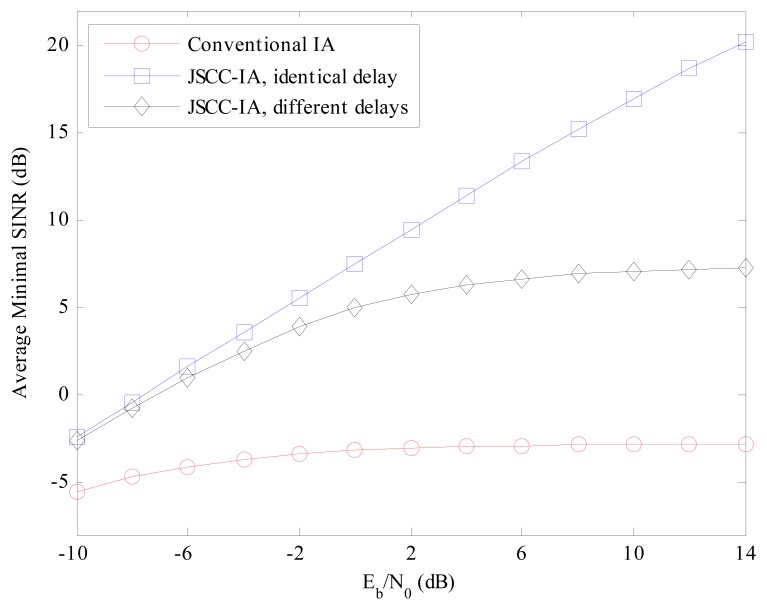
The average minimal received SINR of the conventional IA scheme and the JSCC-IA schemes with identical/different propagation delays in the (6 × 6, 2)^18^ symmetric networks.

Error rate is not the only performance measure of the system, and we also evaluate the schemes from the perspective of minimal received SINR, which represents the performance of the worst data stream. If the minimal received SINR is too low, the communications of some users might be terminated. Therefore, it is important to guarantee the minimal received SINR so that all the users in the network can transmit data normally. The average minimal received SINR of different schemes are compared in Figures 7 and 8 for the (4 × 4, 1)^20^ and (6 × 6, 2)^18^ symmetric networks, respectively.

For the (4 × 4, 1)^20^ network in Figure 7, the minimal received SINR of the conventional IA system increases slowly with the raise in *E_b_*/*N*_0_, and ranges only from −6 to −3 dB, implying that the worst data stream is suffering from strong interference and the communication of the corresponding user is likely to be terminated. On the contrary, the JSCC-IA scheme with identical propagation delay can achieve much higher minimal received SINR, and can reach the value of 17 dB at *E_b_*/*N*_0_ = 14 dB. When different propagation delays are considered, the JSCC-IA scheme might be degraded in the high *E_b_*/*N*_0_ regimes, and cannot reach such a high level as the one with the same delay. In spite of this, it still exhibits much better minimal received SINR than the conventional IA system, with as much as 9 dB higher minimal received SINR than the traditional IA system at *E_b_*/*N*_0_ = 14 dB.

For the multi-data-stream scenario of (6 × 6, 2)^18^ in Figure 8, similar performances can be observed. Considering the *E_b_*/*N*_0_ of 14 dB, the JSCC-IA scheme shows implicit advantage over the conventional IA system, with 23 and 10 dB higher minimal SINR than the traditional one in the cases of identical and different propagation delays, respectively. Therefore, the JSCC-IA scheme can enhance the minimal SINR, and thus reduce the outage probability in the large-scale WSNs.

**Figure 9.**
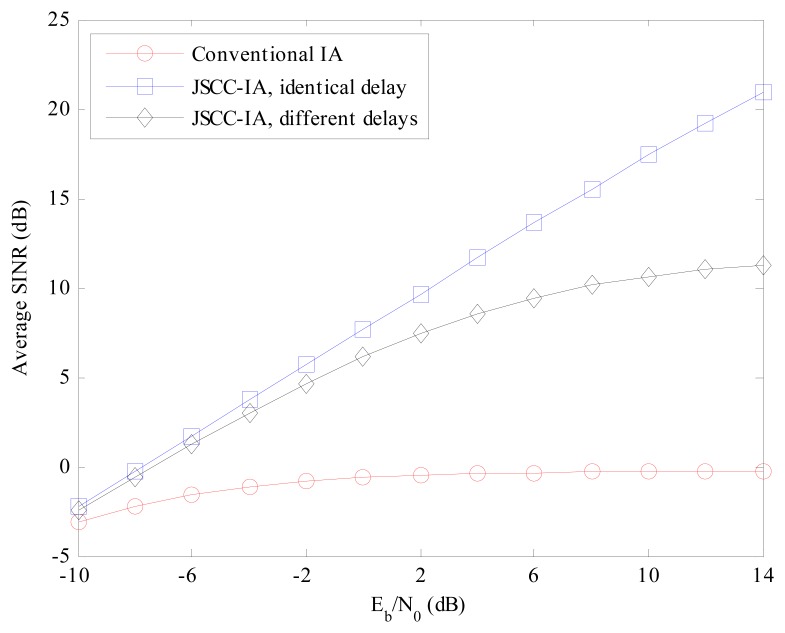
The average received SINR of the conventional IA scheme and the JSCC-IA schemes with identical/different propagation delays in the (4 × 4, 1)^20^ symmetric networks.

**Figure 10.**
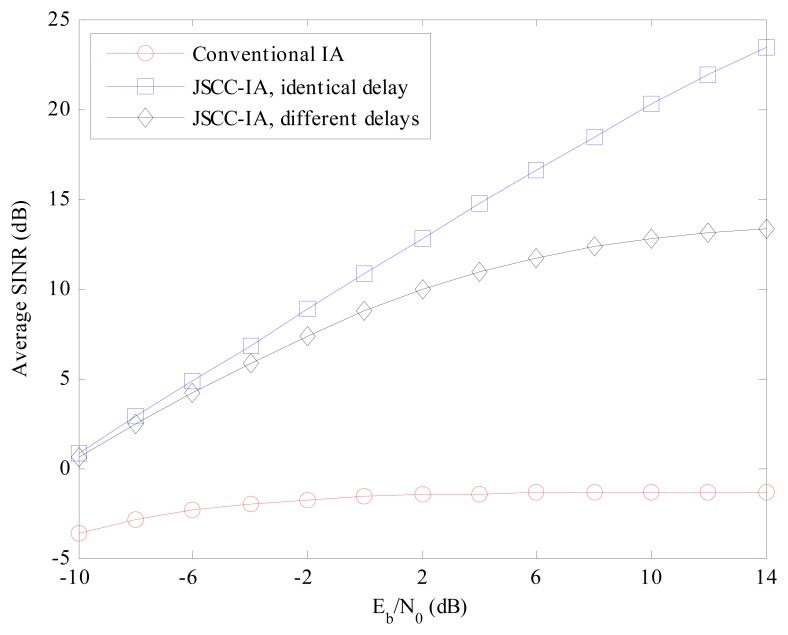
The average received SINR of the conventional IA scheme and the JSCC-IA schemes with identical/different propagation delays in the (6 × 6, 2)^18^ symmetric networks.

Although the minimal received SINR provides us the measure of the performance of the worst data stream, it does not present further details on the other streams. Therefore, we introduce the average received SINR among all the data streams to further evaluate the overall performance thoroughly in Figures 9 and 10.

For the (4 × 4, 1)^20^ network in Figure 9, the performance of the conventional IA scheme is poor, only ranging from the value of −3 to 0 dB from the *E_b_*/*N*_0_ of −10 to 14 dB. Therefore, most of the users in the traditional IA system are suffering from strong interference, and might fail to communicate normally under such a low received SINR level.

However, the JSCC-IA scheme can achieve higher average received SINR, especially in the high *E_b_*/*N*_0_ regimes. For instance, given *E_b_*/*N*_0_ = 14 dB, the average received SINR of the conventional IA scheme, the JSCC-IA scheme with identical propagation delay and the JSCC-IA scheme with different propagation delays is 0 dB, 21 dB and 11 dB, respectively.

For the (6 × 6, 2)^18^ network in Figure 10, the performance of the conventional IA system becomes even poorer due to the larger number of total data streams, and the best average received SINR is only −1 dB at *E_b_*/*N*_0_ = 14 dB, indicating that the communication might be terminated. Meanwhile, the JSCC-IA scheme does not seem to be degraded by the increase in DoFs and the number of total data streams, and still enjoys high average received SINR.

### 6.2. Performance in the Asymmetric Networks

In the simulations above, the users are assumed to be free from large-scale fading. However, in practical communication systems, different users might locate randomly and suffer from different levels of large-scale fading. In this section, we evaluate the IA schemes in more practical asymmetric networks. It is assumed that the transmitters are uniformly located in a 10 km × 10 km square with the receivers randomly distributed around the associated transmitters. The distance between each transmitter-receiver pair is 1 km, and the path-loss exponent α is set to be 3.76 [13]. Different from the simulations in Subsection 6.1, the grouping combinations in the asymmetric networks might have an impact on the performance of the JSCC-IA scheme. Since brute-force searching of all the possible grouping combinations is unacceptable for the networks with large number of users, the RGS JSCC-IA scheme is introduced in Section 5, which can reduce the computational complexity and select better grouping combinations. In this subsection, we evaluate the RGS JSCC-IA scheme and compare it with the conventional IA system. The preset objective minimal received SINR and maximal number of iterations for the RGS JSCC-IA scheme are set to be ξ = 15 dB and as Θ = 20, respectively.

**Figure 11.**
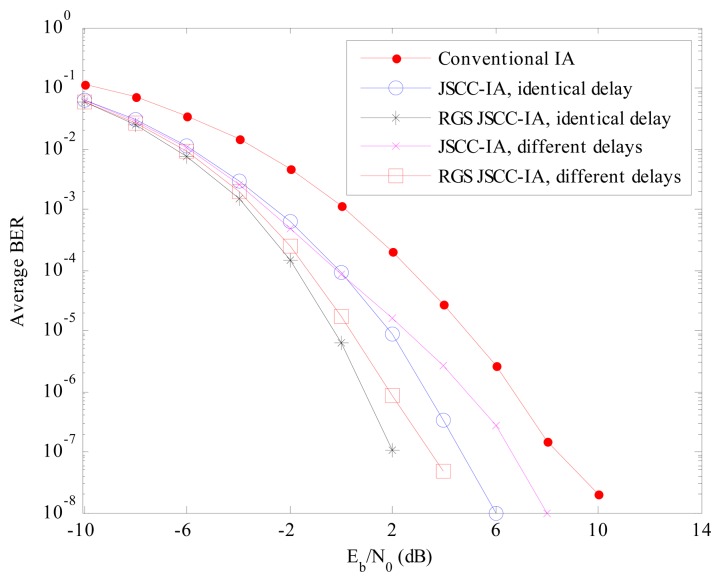
The average experimental BER performances of the conventional IA scheme, the JSCC-IA schemes with identical/different propagation delays and the RGS JSCC-IA schemes with identical/different propagation delays in the (4 × 4, 1)^20^ asymmetric networks.

**Figure 12.**
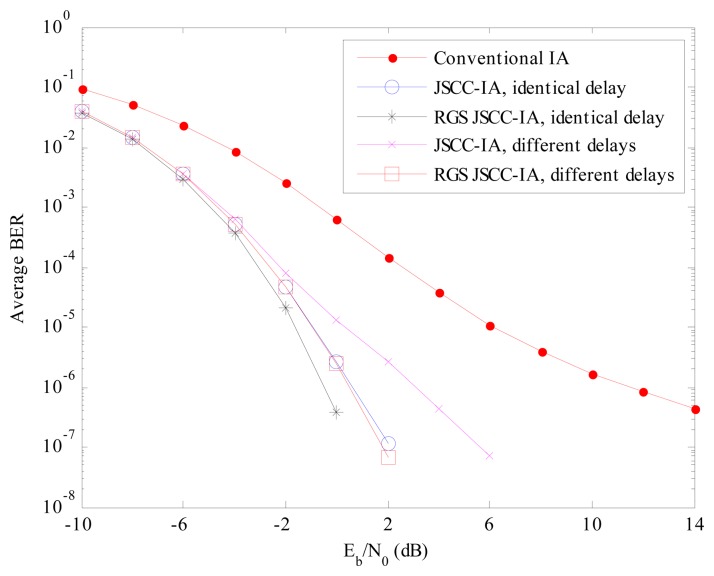
The average experimental BER performances of the conventional IA scheme, the JSCC-IA schemes with identical/different propagation delays and the RGS JSCC-IA schemes with identical/different propagation delays in the (6 × 6, 2)^18^ asymmetric networks.

The error performances of the conventional IA, JSCC-IA and RGS JSCC-IA schemes are compared in Figures 11 and 12 for the (4 × 4, 1)^20^ and (6 × 6, 2)^18^ networks, respectively. From the results, we can observe that the conventional IA system can achieve better BER in the asymmetric than the symmetric networks for the reason that the interference is relatively weaker due to the large-scale fading. In spite of this, the proposed JSCC-IA scheme can still achieve lower average BER than the conventional IA system.

For the (4 × 4, 1)^20^ network in Figure 11, given the BER of 10^−5^, the *E_b_*/*N*_0_ required by the JSCC-IA system with identical propagation delay is 0 dB, compared with the 4 dB required by the conventional IA system. When RGS algorithm is applied, it provides approximately 2 dB *E_b_*/*N*_0_ gains over the non-RGS JSCC-IA scheme at the BER of 10^−5^, and the gain increases with the decrease in BER. Although the different propagation delays degrade the performance of the JSCC-IA system, the difference of the *E_b_*/*N*_0_ between the JSCC-IA schemes with identical and different delays is no more than 2 dB at the same BER. Besides, the JSCC-IA scheme with different propagation delays still outperforms the convention IA system, especially when the RGS algorithm is applied. At *E_b_*/*N*_0_ = 2 dB, the BER of the conventional IA scheme, the RGS JSCC-IA schemes with identical and different propagation delays is 10^−4^, 10^−7^ and 10^−6^, respectively.

For the (6 × 6, 2)^18^ case in Figure 12, the BER performance of the JSCC-IA scheme is better than that of the conventional IA system. Considering the *E_b_*/*N*_0_ of 2 dB, the BER of the JSCC-IA schemes with identical and different propagation delays is respectively 10^−7^ and 10^−6^, while the conventional IA system can only reach the level of 10^−4^. When the RGS algorithm is applied, the RGS JSCC-IA scheme can show even better performances. Given the BER of 10^−8^, the RGS strategy can provide 4 dB *E_b_*/*N*_0_ gains over the non-RGS JSCC-IA scheme for the scenario of different propagation delays. Moreover, the gain increases with the raise in *E_b_*/*N*_0_, implying that the RGS algorithm can select better grouping combinations.

**Figure 13.**
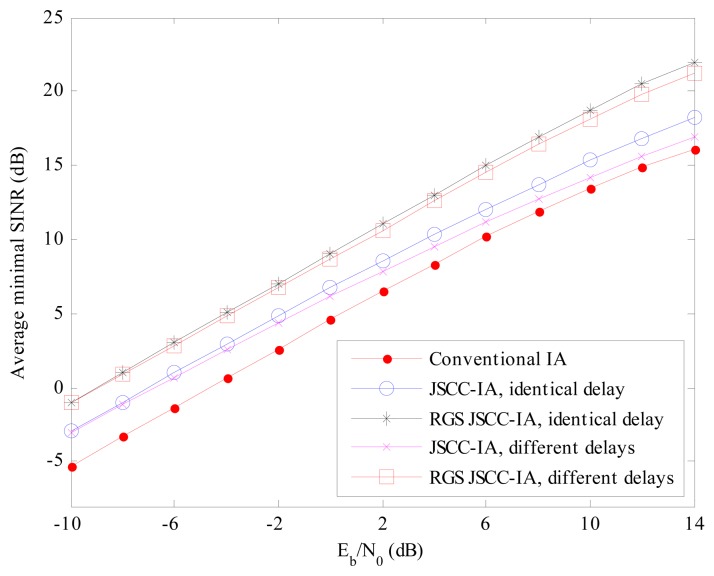
The average minimal received SINR of the conventional IA scheme, the JSCC-IA schemes with identical/different propagation delays and the RGS JSCC-IA schemes with identical/different propagation delays in the (4 × 4, 1)^20^ asymmetric networks.

Like the symmetric networks, we also evaluate the schemes from the perspective of minimal received SINR in order to evaluate the performance of the worst data stream. For the (4 × 4, 1)^20^ network in Figure 13, the JSCC-IA scheme with identical propagation delay can achieve about 2 dB higher minimal received SINR than the conventional IA system at the same *E_b_*/*N*_0_. The RGS algorithm can further improve the performance, and provide an extra gain of 2 to 4 dB over the non-RGS JSCC-IA scheme from the low to high *E_b_*/*N*_0_ regimes. The difference between the JSCC-IA schemes with identical and different propagation delays is small at low *E_b_*/*N*_0_ regimes, and is no more than 1.5 dB at the high *E_b_*/*N*_0_ regimes. Similarly, for the RGS JSCC-IA schemes, the different propagation delays do not have a significant impact on the minimal received SINR, and only yield slight degradation in the high *E_b_*/*N*_0_ regimes.

**Figure 14.**
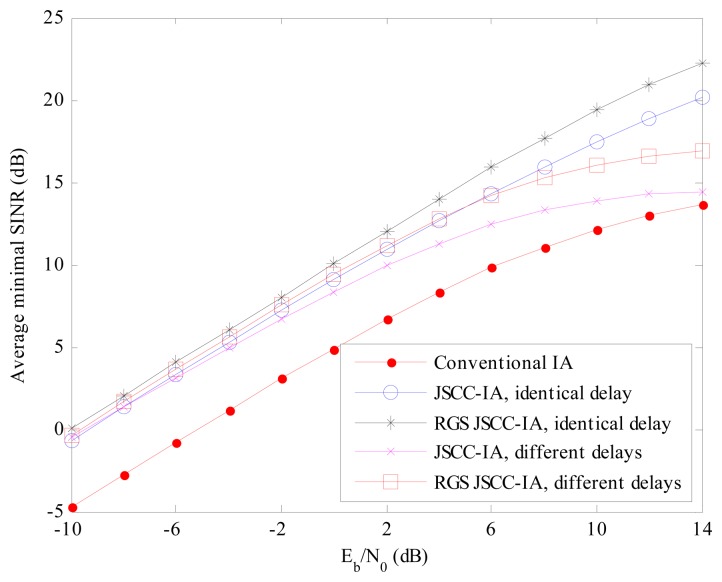
The average minimal received SINR of the conventional IA scheme, the JSCC-IA schemes with identical/different propagation delays and the RGS JSCC-IA schemes with identical/different propagation delays in the (6 × 6, 2)^18^ asymmetric networks.

For the (6 × 6, 2)^18^ network in Figure 14, the total number of data steams is 36, which is much larger than that of the (4 × 4, 1)^20^ network. The minimal received SINR of the conventional IA system is further degraded by the stronger interference, and the best minimal received SINR is only 14 dB at *E_b_*/*N*_0_ =14dB.

On the contrary, the JSCC-IA scheme with identical propagation delay can achieve much higher minimal received SINR, and exhibits a gain of 4 to 6 dB over the conventional IA system from the low to high *E_b_*/*N*_0_ regimes. Moreover, the RGS algorithm can further improve the performance, and achieve about 2 dB higher minimal received SINR than the non-RGS JSCC-IA scheme with identical delay at *E_b_*/*N*_0_ =14 dB. Although the performance of the JSCC-IA scheme with different propagation delays is inferior to that with the same delay, it still shows higher minimal received SINR than the conventional IA system. In addition, the RGS strategy can improve the performance, and the minimal received SINR of the RGS JSCC-IA system with different delays at *E_b_*/*N*_0_ =14 dB is 17 dB, which is about 3 dB higher than that of the conventional IA system. Therefore, the proposed JSCC-IA and RGS JSCC-IA schemes can improve the performance of the worst data stream and reduce of the chance of communication outage.

**Figure 15.**
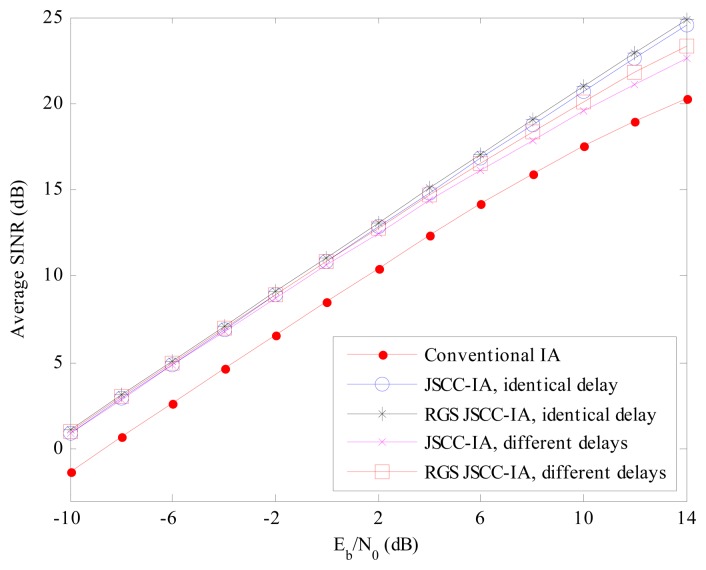
The average received SINR of the conventional IA scheme, the JSCC-IA schemes with identical/different propagation delays and the RGS JSCC-IA schemes with identical/different propagation delays in the (4 × 4, 1)^20^ asymmetric networks.

In addition, we also compare the average received SINR in order to provide a more thorough insight into the JSCC-IA schemes. For the (4 × 4, 1)^20^ network in Figure 15, the average received SINR of the JSCC-IA and RGS JSCC-IA schemes have almost the same value in the low *E_b_*/*N*_0_ regimes, and they exhibit about 2 dB higher average received SINR over the conventional IA system under the same *E_b_*/*N*_0_. On the other hand, when *E_b_*/*N*_0_ increases, the proposed schemes show even better performances. Considering *E_b_*/*N*_0_ = 14 dB, the RGS JSCC-IA schemes with identical and different propagation delays can provide about 4 and 3 dB higher average SINR than the conventional IA system, respectively.

**Figure 16.**
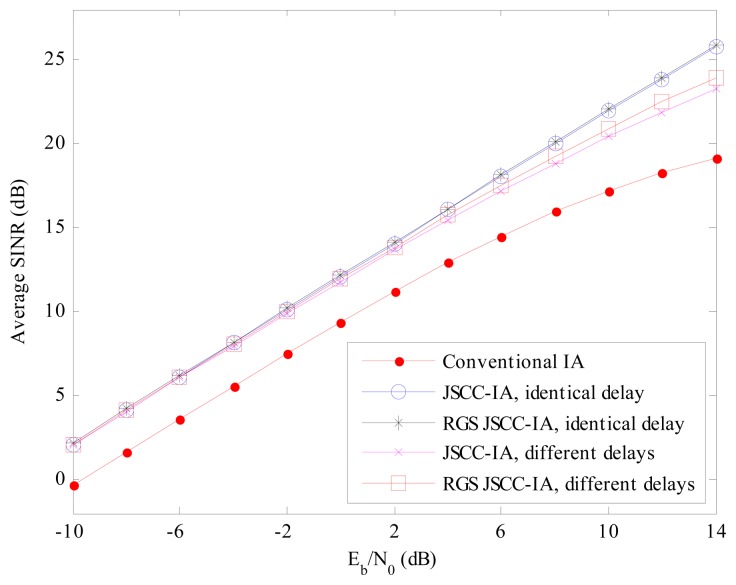
The average received SINR of the conventional IA scheme, the JSCC-IA schemes with identical/different propagation delays and the RGS JSCC-IA schemes with identical/different propagation delays in the (6 × 6, 2)^18^ asymmetric networks.

For the (6 × 6, 2)^18^ network in Figure 16, similar performances can be observed. In the low *E_b_*/*N*_0_ regimes, the proposed schemes can achieve about 2.5 dB higher average received SINR than the convention IA scheme. Moreover, the difference of average received SINR between the conventional and the proposed schemes increases with the rise in *E_b_*/*N*_0_. For instance, the average SINR at *E_b_*/*N*_0_ = 14 dB is 19 dB, 26 dB and 24 dB for the conventional IA scheme, the (RGS) JSCC-IA schemes with identical delay and the (RGS) JSCC-IA schemes with different delays, respectively. Therefore, simulations validate that the proposed JSCC-IA schemes can improve the overall performance of the networks by increasing the averaged received SINR.

To sum up, the proposed JSCC-IA scheme can accomplish better BER and received SINR performances than the conventional IA system, not only in symmetric but also in asymmetric networks. Moreover, the RGS algorithm can search for better grouping combinations and provide further improvement over the non-RGS JSCC-IA scheme in the asymmetric networks.

## 7. Conclusions and Future Work

In this paper, a novel joint spatial-code clustered IA scheme has been proposed to mitigate the strong interference that cannot be suppressed well by the traditional IA system in large-scale wireless sensor networks with limited antennas. In the proposed JSCC-IA scheme, the users are clustered into several groups, and traditional IA methods such as the Max-SINR algorithm are applied within each group to eliminate the intra-group interference. Moreover, each group is assigned with an exclusive PN code so than the inter-group interference can be mitigated through the orthogonality of the PN codes. The analytical BER expressions of the the proposed JSCC-IA scheme have been formulated in the situations of identical and different propagation delays, respectively. Both of the symmetric and asymmetric networks have been considered, and a random selection grouping algorithm has been introduced to search for better grouping combinations for the latter circumstance. Numerical results have demonstrated that the proposed JSCC-IA scheme outperforms the conventional IA system, and can achieve significantly lower BER and higher received SINR in the situation where the number of users exceeds the IA feasibility constraint.

Perfect CSI is assumed to be available and the signaling feedback strategy is not discussed in this paper. However, in the practical WSNs, the ideal CSI might be difficult to acquire due to the feedback latency, estimation error and so on. In our future work, we will extend our work to the scenario of imperfect CSI to make the proposed scheme more practical. Moreover, we will further investigate the relationship between the grouping combinations and the error performance. We will develop a more intelligent and efficient clustering algorithm to search for even better grouping combinations.

## Figures and Tables

**Table 1.** The number of overall complex multiplications required by the RGS and BFS algorithms.

**(*M*,*K*,*d*,*K*_0_,*G*)**	***C*_BFS1_**	***C*_BFS2_**	***C*_RGS1_**	***C*_RGS2_**	***C*_RGS1_/*C*_BFS1_**	***C*_RGS2_/*C*_BFS2_**
(2,6,1,2,3)	5.51×10^4^	7.94 × 10^4^	1.22 × 10^4^	1.76 × 10^4^	2.22 × 10^−1^	2.22 × 10^−1^
(2,8,1,2,4)	2.42 × 10^6^	3.69 × 10^6^	1.92 × 10^4^	2.93 × 10^4^	7.94 × 10^−3^	7.94 × 10^−3^
(2,10,1,2,5)	1.56 × 10^8^	2.48 × 10^8^	2.76 × 10^4^	4.38 × 10^4^	1.76 × 10^−4^	1.76 × 10^−4^
(4,10,1,5,2)	2.98 × 10^6^	3.50 × 10^6^	2.36 × 10^5^	2.78 × 10^5^	7.94 × 10^−2^	7.94 × 10^−2^
(4,15,1,5,3)	1.47 × 10^10^	1.84 × 10^10^	3.89 × 10^5^	4.86 × 10^5^	2.64 × 10^−5^	2.64 × 10^−5^
(4,20,1,5,4)	3.31 × 10^14^	4.34 × 10^14^	5.65 × 10^5^	7.40 × 10^5^	1.70 × 10^−9^	1.70 × 10^−9^
(4,25,1,5,5)	2.38 × 10^19^	3.24 × 10^19^	7.63 × 10^5^	1.04 × 10^6^	3.21 × 10^−14^	3.21 × 10^−14^
(4,8,2,2,4)	5.06 × 10^7^	6.36 × 10^7^	4.02 × 10^5^	5.05 × 10^5^	7.94 × 10^−3^	7.94 × 10^−3^
(4,10,2,2,5)	3.06 × 10^9^	4.00 × 10^9^	5.39 × 10^5^	7.05 × 10^5^	1.76 × 10^−4^	1.76 × 10^−4^

## Appendix

### Proof of Theorem 1

For convenience, the vectors **n̂***_k_* and **n***_k_*(*t*) are first expanded as follows:

(A.1) $${\hat{n}}_{k}={\left[{\hat{n}}_{k,1},{\hat{n}}_{k,2},\ldots ,{\hat{n}}_{k,N}\right]}^{\text{T}},n_{k}(t)={\left[n_{k,1}(t),n_{k,2}(t),\ldots ,n_{k,N}(t)\right]}^{\text{T}}$$

From Equations (11) and (32), the *l*-th component of **n̂***_k_* can be expressed as:

(A.2) $${\hat{n}}_{k,l}=\int_{0}^{T_{b}}U_{k,l}^{\text{H}}n_{k}(t)g_{k}(t)dt=\int_{0}^{T_{b}}\overset{N}{\underset{j=1}{\text{∑}}}U_{k}{\left(j,l\right)}^{*}n_{k,j}(t)g_{k}(t)dt.$$

Since *n_k_*,*_j_*(*t*) is a zero-mean AWGN process, the expected value of *n̂_k,l_* can be expressed as:

(A.3) $$E[{\hat{n}}_{k,l}]=\int_{0}^{T_{b}}\overset{N}{\underset{j=1}{\text{∑}}}U_{k}{\left(j,l\right)}^{*}E[n_{k,j}(t)g_{k}(t)]dt=0$$

The variance of *n̂_k,l_* can be expressed as:

(A.4) $$\begin{array}{ll} \mathrm{var}[{\hat{n}}_{k,l}] & =E[{\left|{\hat{n}}_{k,l}-E[{\hat{n}}_{k,l}]\right|}^{2}]=E[{\hat{n}}_{k,l}{\hat{n}}_{k,l}^{*}]\\ & =E[\int_{0}^{T_{b}}\overset{N}{\underset{j=1}{\text{∑}}}U_{k}{\left(j,l\right)}^{*}n_{k,j}(t)g_{k}(t)dt\int_{0}^{T_{b}}\overset{N}{\underset{p=1}{\text{∑}}}U_{k}(p,l)n_{k,p}^{*}(\tau )g_{k}(\tau )d\tau ]\\ & =\overset{N}{\underset{j=1}{\text{∑}}}\overset{N}{\underset{p=1}{\text{∑}}}U_{k}{\left(j,l\right)}^{*}U_{k}(p,l)\int_{0}^{T_{b}}\int_{0}^{T_{b}}E[n_{k,j}(t)n_{k,p}^{*}(\tau )]g_{k}(t)g_{k}(\tau )\text{dtd}\tau \end{array}$$

When *j* ≠ *p*, *n_k_*,*_j_*(*t*) and *n_k_*,*_p_*(*t*) are uncorrelated and we have:

(A.5) $$E[n_{k,j}(t)n_{k,p}^{*}(\tau )]=0,j\neq p$$

When *j* = *k*, as *n_k_*,*_j_*(*t*) is an AWGN process with two-sided power spectral density of *N*_0_/2 W/Hz, we have:

(A.6) $$E[n_{k,j}(t)n_{k,j}^{*}(\tau )]=\frac{N_{0}}{2}\delta (t-\tau )$$

Substitute Equations (A.5) and (A.6) into Equation (A.4), and the variance of *n̂_k,l_* can be written as:

(82) $$\begin{array}{ll} \mathrm{var}[{\hat{n}}_{k,l}] & =\overset{N}{\underset{j=1}{\text{∑}}}U_{k}{\left(j,l\right)}^{*}U_{k}(j,l)\int_{0}^{T_{b}}\frac{N_{0}}{2}g_{k}(t)g_{k}(t)dt\\ & =\frac{N_{0}}{2}E_{b}\overset{N}{\underset{j=1}{\text{∑}}}|{\left|U_{k}(j,l)\right|}^{2}\\ & =N_{0}E_{b}/2 \end{array}$$

In addition, the real and imaginary parts of *n̂_k,l_* share the same distribution, and the expected value and variance of the real part of *n̂_k,l_* can be calculated as:

(83) $$E[\text{real}({\hat{n}}_{k,l})]=E[{\hat{n}}_{k,l}]/2=0$$

(84) $$\mathrm{var}[\text{real}({\hat{n}}_{k,l})]=\mathrm{var}[{\hat{n}}_{k,l}]/2=N_{0}E_{b}/4$$
